# Myocardial and haemodynamic responses to two fluid regimens in African children with severe malnutrition and hypovolaemic shock (AFRIM study)

**DOI:** 10.1186/s13054-017-1679-0

**Published:** 2017-05-03

**Authors:** Nchafatso Obonyo, Bernadette Brent, Peter Olupot-Olupot, Michael Boele van Hensbroek, Irene Kuipers, Sidney Wong, Kenji Shiino, Jonathan Chan, John Fraser, Job B. M. van Woensel, Kathryn Maitland

**Affiliations:** 1 KEMRI-Wellcome Trust Research Programme, Centre for Geographic Medicine Research-Coast, Kilifi, Kenya; 2 Wellcome Trust Centre for Clinical Tropical Medicine and Department of Paediatrics, Faculty of Medicine, Imperial College, London, W2 1PG UK; 3 Department of Paediatrics, Mbale Regional Referral Hospital, Mbale, Uganda; 4 Department of Global Health and Department of Paediatrics, Academic Medical Centre, University of Amsterdam, Amsterdam, Netherlands; 5 Médecins Sans Frontières - Operational Centre Amsterdam, Plantage Middenlaan 14, 1018 DD Amsterdam, Netherlands; 6 Critical Care Research Group, The Prince Charles Hospital, Brisbane, Queensland, Australia; 7 School of Medicine, Griffith University, Nathan, Queensland Australia

**Keywords:** Severe malnutrition, African, Children, Hypovolaemic shock, Gastroenteritis, Mortality, Myocardial function, Echocardiography, Fluid resuscitation, Ringers lactate

## Abstract

**Background:**

Fluid therapy in severely malnourished children is hypothesized to be deleterious owing to compromised cardiac function. We evaluated World Health Organization (WHO) fluid resuscitation guidelines for hypovolaemic shock using myocardial and haemodynamic function and safety endpoints.

**Methods:**

A prospective observational study of two sequential fluid management strategies was conducted at two East African hospitals. Eligible participants were severely malnourished children, aged 6–60 months, with hypovolaemic shock secondary to gastroenteritis. Group 1 received up to two boluses of 15 ml/kg/h of Ringer’s lactate (RL) prior to rehydration as per WHO guidelines. Group 2 received rehydration only (10 ml/kg/h of RL) up to a maximum of 5 h. Comprehensive clinical, haemodynamic and echocardiographic data were collected from admission to day 28.

**Results:**

Twenty children were enrolled (11 in group 1 and 9 in group 2), including 15 children (75%) with kwashiorkor, 8 (40%) with elevated brain natriuretic peptide >300 pg/ml, and 9 (45%) with markedly elevated median systemic vascular resistance index (SVRI) >1600 dscm-^5^/m^2^ indicative of severe hypovolaemia. Echocardiographic evidence of fluid-responsiveness (FR) was heterogeneous in group 1, with both increased and decreased stroke volume and myocardial fractional shortening. In group 2, these variables were more homogenous and typical of FR. Median SVRI marginally decreased post fluid administration (both groups) but remained high at 24 h. Mortality at 48 h and to day 28, respectively, was 36% (4 deaths) and 81.8% (9 deaths) in group 1 and 44% (4 deaths) and 55.6% (5 deaths) in group 2. We observed no pulmonary oedema or congestive cardiac failure on or during admission; most deaths were unrelated to fluid interventions or echocardiographic findings of response to fluids.

**Conclusion:**

Baseline and cardiac response to fluid resuscitation do not indicate an effect of compromised cardiac function on response to fluid loading or that fluid overload is common in severely malnourished children with hypovolaemic shock. Endocrine response to shock and persistently high SVRI post fluid-therapy resuscitation may indicate a need for further research investigating enhanced fluid volumes to adequately correct volume deficit. The adverse outcomes are concerning, but appear to be unrelated to immediate fluid management.

**Electronic supplementary material:**

The online version of this article (doi:10.1186/s13054-017-1679-0) contains supplementary material, which is available to authorized users.

## Background

The World Health Organization (WHO) severe malnutrition guidelines for treatment of hypovolaemic shock recommend reserving intravenous fluids for those presenting with advanced shock, and recommend low-volume hypotonic fluids [1]. These recommendations are founded on the assumption that the “malnourished” heart is at risk of incipient biventricular failure and thus unable to respond to isotonic fluid challenges; however, this remains controversial owing to the weak evidence base, including randomised clinical trials (RCTs) or appropriate physiological studies [2–4].

A study conducted in Bangladesh in which standard fluid resuscitation/rehydration was used in malnourished children showed no evidence of heart failure in the marasmic children [5]. A phase II randomised trial in which half-strength (hypotonic) Darrow’s/5% dextrose (usual care) was compared to isotonic Ringer’s lactate (RL) in severely malnourished Kenyan children with shock demonstrated superior shock reversal and lower mortality in the RL arm and supported further research examining the rate and volume of fluid replacement [2]. However, publication of a multicentre RCT, (Fluid Expansion as A Supportive Therapy trial (FEAST)) [6], which demonstrated increased mortality in the fluid bolus arms compared to control (no-bolus) in African children with severe febrile illness, has necessitated further research to elucidate the mechanism of harm with fluid boluses. Children with shock due to gastroenteritis were excluded from the FEAST trial, thus, further research is required to generate evidence for best practice in this group.

Severe dehydrating diarrhoea is a common complication of severe malnutrition, as demonstrated in a prospective study of severely malnourished Kenyan children, in which >50% of patients admitted had this complication, with an associated overall 20% fatality rate, increasing to 38% in the presence of severe dehydration and delayed capillary refill time [7]. Most deaths occur early in the course of admission [8]. The high mortality in this sub-group and lack of relevant research to inform management guidelines warranted this prospective study in children with severe malnutrition, to examine myocardial function and haemodynamic response to fluid resuscitation in hypovolaemic shock due to gastroenteritis (the Appropriate Fluid Resuscitation In Malnutrition (AFRIM) study).

## Methods

### Study location and participants

The study was conducted between May 2013 and August 2014 at the Kilifi District Hospital (KDH), Kenya and the Mbale Regional Referral Hospital (MRRH), Uganda. Eligible patients included children aged 6–60 months with clinical signs of severe malnutrition (defined as any one of the following: mid-upper arm circumference (MUAC) <11.5 cm, weight-for-height Z score (WHZ) below –3 or oedema indicative of kwashiorkor plus acute hypovolaemic diarrhoea (>3 watery stools/24 h) with signs of severe dehydration (sunken eyes and/or decreased skin turgor) and shock. We defined shock as two or more signs of capillary refill time ≥3 seconds, temperature gradient (cooler extremities to warmer central body to touch) or rapid and weak pulse volume, instead of the WHO stringent shock definition (which includes all four features of impaired perfusion plus impaired consciousness) [1], which identifies very few children with extremely poor prognosis [8]. We excluded children with severe dermatitis of the groin (in whom catheterization for urine output monitoring was not possible), children with congenital heart disease and children whose guardians did not consent to study participation.

### Study procedures

Written informed consent was obtained from the children’s guardians once the patients had been stabilised following initial verbal assent at the start of resuscitation [9]. Eligible and consenting participants were recruited sequentially. The bolus group (group 1) followed WHO standard protocol, which recommends a bolus of 15 ml/kg of RL over 1 h, with the option of repeating it once (15 ml/kg) if signs of shock persist [1], followed by intravenous half-strength Darrow’s/5% dextrose (HSD/5D) given at a rate of 4 ml/kg/h.

Following a pre-planned interim analysis of the clinical data after 10 patients were enrolled (which did not include detailed review of the echocardiographic and haemodynamic data) the study investigators were very concerned about the very high mortality rate. In light of the original findings and emerging published data from the FEAST trial [6, 10], the research team, concerned that the bolus fluid challenges could be leading to compromised myocardial and haemodynamic responses, opted to prospectively study a further 10 patients who would receive rehydration volume replacement without an initial fluid bolus. The rehydration-only group (group 2) received 10 ml/kg/h of RL over 5 h.

In both groups children were switched to oral rehydration (rehydration solution for malnutrition, ReSoMal) once they were able to tolerate oral intake or nasogastric fluids. Blood transfusion, if indicated by WHO guidelines (haemoglobin (Hb) <5 g/dl or shock unresponsive to crystalloid volume replacement), was administered as 10 ml/kg over 3 h once blood for transfusion was available. All the patients received routine standard treatments for severe malnutrition, as per the WHO guidelines, including antibiotics and therapeutic formula feeding once able to tolerate oral feeds [1].

### Clinical assessment

Patients underwent the standard admission procedures including clinical examination and routine blood sampling. Blood was stored for retrospective analysis of biomarkers of myocardial injury (cardiac troponin I (cTnI)) or congestive heart failure (brain natriuretic peptide (BNP)) based on published literature [11–15]. At each clinical review routine vital-status and physical examination parameters were systematically recorded and assessed for key endpoints (shock reversal, adverse events related to fluid overload including evidence of pulmonary oedema (bi-basal crepitations and worsening oxygen saturations), raised jugular venous pressure, gallop rhythm and increasing hepatomegaly). Electrocardiogram (ECG) and echocardiogram (ECHO) were used to assess cardiac function both pre and post resuscitation (immediately and at 24 h) and at one-month follow up.

### Echocardiography measurements

All patients were studied using a *Vivid.i* portable ultrasound device (General Electric Medical Systems ®, USA) with simultaneous ECG display. A rapid haemodynamic assessment was conducted at admission and a comprehensive ECHO was performed when the patient was stabilised. Three echocardiographic recordings and readings were obtained during three consecutive cardiac cycles and an average of these was used to evaluate the baseline status and the response to fluid therapy. A standardized protocol was used for ECHO measurement of left ventricular (LV) size and function, which was in accordance with the guidelines published by the American Society of Echocardiography, updated in 2014 [16]. Additional file 1: Box 1 presents the details of the calculations.

### Electrocardiography measurements

A standard twelve-lead ECG (25 mm/sec) was recorded in all patients at admission, 24 h post resuscitation and during follow up (Cardiac Acquisition Module, CAM-14, GE Medical Systems and CardioSoft ™ software). Collected data included: duration of P wave, PR interval, duration of QRS complex, RR interval, PP interval and QT interval. Corrected QT interval (QTc) was calculated using Bazett’s formula [17, 18].

### Data analysis

Data were analysed using STATA 12 software (STATA ® Corp, United States of America). We report descriptive statistics including median and interquartile ranges for pooled measures of cardiac function, haemodynamic response and blood parameters with comparison between groups 1 and 2. Cut-off values for normality were based on previously published data [19–22]. In addition, we summarised the haemodynamic profiles of the children (heart rate, systolic and diastolic blood pressure) across the time point (0–48 h) using age-adjusted medians stratified by group and survival status. Kaplan-Meier analyses were used for comparison of time to mortality between the two groups. The exact 95% confidence intervals were evaluated using the log-rank test for equality of survivor functions. The significance level was 0.05 for all statistical tests conducted. No multivariate analyses were conducted owing to the small sample size.

## Results

### Recruitment

**Fig. 1:**
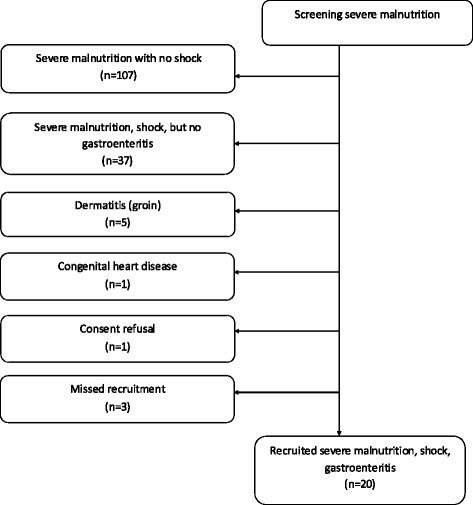
AFRIM study flow

A total of 174 severely malnourished patients were screened during the study period (Fig. 1). Of these, 107 did not have any signs of shock, 37 had signs of shock that was not secondary to gastroenteritis (septic shock), 5 had severe dermatitis in the groin, 1 had a congenital cardiac anomaly, 1 refused consent, 2 had already received fluid boluses and 1 presented outside the study daily recruitment time. A total of 20 patients with hypovolaemic shock and gastroenteritis were prospectively recruited, including 11 in group 1 (bolus followed by rehydration), and 9 in group 2 (rehydration-only fluid).

### Baseline data

The overall median age in the two groups was 21.5 months (interquartile range 17, 25.5) with no difference between the cohorts (Table 1). More patients with oedematous or kwashiorkor phenotype (10/11; 91%) were enrolled in group 1 compared to group 2 (5/9; 56%). This was possibly due to the seasonal nature of this complication. Baseline clinical and haemodynamic parameters were similar in the two groups. Overall, respiratory signs were very prevalent including tachypnoea, respiratory distress and hypoxia (oxygen saturations <90% in room air on pulse oximetry). As expected features of shock, dehydration and impaired consciousness were common, but only 35% of patients (n = 7) fulfilled the strict WHO criteria for shock. Besides hypoglycaemia (<3 mmol/L), which was more common in group 2, there were no significant differences between the groups at baseline (Table 1). The large proportion of patients with malaria parasitaemia reflects the background endemic malaria in Eastern Uganda. Haemoglobin levels were low; but only two patients had severe anaemia (Hb <5 g/dl).

**Table 1:** Baseline characteristics

Category	Feature present at admission	Group 1 Bolus + rehydration		Group 2 Rehydration only		*P* value
Total number		N	11	n	9	N/A
Malnutrition type	Oedematous (Kwashiorkor)	10	(90.9%)	5	(55.6%)	0.08
Sex	Male (%)	5	(45.5%)	7	(77.8%)	0.15
Age	Median age (months)	21	(12–43)	24	(9–49)	0.65
Anthropometry	Median weight (kg)	6.1	(5–10)	6	(4–10)	0.91
	Median MUAC (cm)	11.5	(8.5–12.4)	10.2	(7–12.3)	0.20
Clinical	Fever (axillary temperature >37.5 °C)	0	(0%)	0	(0%)	N/A
	Hypothermia (axillary temperature <35.0 °C)	5	(45.5%)	2	(22.2%)	0.19
	Tachypnoea (respiratory rate >40 breaths/minute)	4	(36.4%)	6	(66.7%)	0.52
	Chest indrawing	10	(100%)	9	(100%)	N/A
	Deep breathing (respiratory distress)	9	(90%)	4	(44.4%)	0.04
	Hypoxia (percutaneous oxygen saturation <90%)	5	(45.5%)	5	(62.5%)	0.45
	Tachycardia^a^	1	(9.1%)	4	(44.4%)	0.09
	Bradycardia (heart rate <80 beats/minute)	1	(9.1%)	1	(11.1%)	0.32
	Prolonged capillary refill time (>3 seconds)	5	(45.5%)	4	(44.4%)	0.59
	Weak pulse volume	7	(63.6%)	6	(66.7%)	0.89
	Temperature gradient	11	(100%)	9	(100%)	N/A
	Decreased skin turgor (dehydration)	6	(54.5%)	7	(77.8%)	0.82
	Prostration or coma	9	(81.8%)	4	(44.4%)	0.18
Shock	WHO shock definition^b^	4	(36.4%)	3	(33.3%)	0.58
Heart failure definition (see Table 2: definitions of heart failure)	Congestive cardiac failure (CCF)	1	(9.1%)	2	(22.2%)	0.70
	Myocardial dysfunction (MD)	2	(18.2%)	1	(11.1%)	0.35
	Elevated brain natriuretic protein (BNP)	6	(54.5%)	2	(22.2%)	0.39
	All three (CCF, MD plus BNP)	1	(9.1%)	1	(11.1%)	0.67
Laboratory	Severe anaemia (haemoglobin <5 g/dL)	2	(22.2%)	0	(0%)	0.27
	Leukocytosis (white blood cell count >12.5 × 10^12^/L)	6	(66.7%)	3	(37.5%)	0.56
	Thrombocytopenia (platelet count <160 × 10^12^/L)	5	(55.6%)	6	(75%)	0.28
	Severe hyponatremia (sodium <125 mmol/L)	4	(36.4%)	5	(62.5%)	0.41
	Severe hypokalemia (potassium <2.5 mmol/L)	5	(50%)	3	(37.5%)	0.37
	Hypoglycaemia (blood glucose <3 mmol/L)	1	(12.5%)	5	(62.5%)	0.02
	Hypercreatinaemia (creatinine >80 μmol/L)	4	(40%)	3	(37.5%)	0.82
	Hyperlacataemia (lactate >3 mmol/L)	3	(33.3%)	3	(33.3%)	N/A
Microbiology/malaria	Malaria parasitaemia (blood film)	5	(55.6%)	3	(42.9%)	0.63

*MUAC* mid-upper arm circumference, *WHO* World Health Organization. ^a^Tachycardia (European Paediatric Life Support): heart rate > 160 beats/minute at age <12 months old; >120 beats/minute at age 12 months to 5 years. WHO shock determined by all four of the following: delayed capillary refill time of >3 sec, weak and fast pulse, cold extremities and lethargy or unconsciousness

Owing to the very limited validation of the non-specific WHO clinical definitions of heart failure (see Table 2) we interpreted these incorporating clinical, echocardiographic and cardiac biomarkers definitions and applied the criteria retrospectively. At baseline they indicated that only 3/20 children (15%) fulfilled the WHO clinical criteria of putative congestive heart failure. However, one patient presented with pneumonic consolidation and two had respiratory distress (Kussmauls breathing) secondary to severe acidosis. None of the children had a gallop rhythm or raised jugular venous pressure (JVP).

**Table 2:** Definitions of heart failure

a) **Clinical**: probable congestive cardiac failure (CCF) scored on a scale ranging from 0 to 5 based on the following signs listed in the WHO guidelines [1]: (1) tachycardia (heart rate >160/minute in a child <12 months of age or >120/minute in a child aged 12 months to 5 years); (2) tachypnoea; (3) gallop heart rhythm; (4) tender hepatomegaly and (5) raised jugular venous pressure seen as distended neck veins. We used a score ≥3 as the cut-off for probable CCF.
b) **Echocardiographic**: myocardial dysfunction (MD) definition was based on the left-ventricular Tei index >0.4 [45–47].
c) **Biochemical**: brain natriuretic protein (BNP) >300 pg/ml [11, 12].

### Interventions

In group 1, 9/11 patients (82%) received two boluses of 15 ml/kg/h RL to correct clinical signs of shock; 4 children (36%) received an additional 10 ml/kg blood transfusion for persistent clinical signs of shock after receiving the RL boluses (in accordance with the current WHO management guideline). One patient presenting with profound anaemia (Hb <2.2 g/dl) received only an initial intravenous RL bolus prior to receiving a blood transfusion. One patient went on to receive oral rehydration following improvement after a single RL bolus at 15 ml/kg/h.

In group 2, 3/9 patients (33%) received 10 ml/kg/h RL rehydration over a total of 5 h (i.e. 50 ml/kg), followed by blood transfusion due to persisting signs of shock (as per the WHO protocol). Four patients (44%) substantially improved after 3 h of intravenous rehydration (30 ml/kg) and were able to tolerate oral ReSoMal rehydration. Two patients died whilst receiving rehydration (at 2 h and 4 h after starting fluid therapy).

### Clinical features over time

Tachypnoea, respiratory distress and the proportion of children with hypoxia did not worsen after fluid therapy in either of the groups. Although hepatomegaly was common at admission there was no evidence of increasing organomegaly complicating fluid resuscitation in any patient. Nor did we find evidence of our pre-specified criteria of fluid overload (Additional file 2: Table S1a and b). Median age-adjusted heart rate and blood pressure over time are presented in Additional file 3: Figure S1a-f. Severe hyponatraemia, present at admission in four patients in group 1 and five patients in group 2 (Table 1), changed little over time, with survivors to day 28 having persisting hyponatraemia at follow up (Additional file 4: Table S2 and Additional file 5: Figure S2). Similarly, median potassium was very low, particularly in group 1 at admission (2.4 mmol/L, IQR = 2.1–3.3 mmol/L) with little improvement over time. High serum creatinine (>80 μmol/L) was present in 4/11 patients (36%) and 3/9 patients (33%) in groups 1 and 2, respectively, which improved gradually over time. Reassuringly, we noted satisfactory urine output (>1 ml/kg/h) in 8/11 patients in group 1 and 7/9 patients in group 2 (Table 3).

**Table 3:** Percentage change in SVI, EDVI, SVRI, IVCCI and FS in individual patients pre and post fluid challenges and total input and output volumes

Patients	Cardiac haemodynamics summary					Fluid resuscitation summary	
	% ∆SVI	% ∆EDVI	% ∆SVRI	% ∆IVCCI	% ∆FS	Input (ml/kg/h)	Output (ml/kg/h)
Group 1							
301	-17	-2	-29	-13	-19	2.8	4.6
401^a^	16	-24	10		66	7.4	1.5
402	-3	-14	20	166	16	3.5	0.7
403	15	-1	-11	118	22	3.4	5.1
404	-1	0	19	29	-2	3.4	6.8
405	33	31	-17	17	2	2.6	7.4
406	-48	-40	56	-16	-18	6.8	0.2
407	57	5	-25	38	60	5.0	0.3
408	46	34	4	-20	13	4.2	1.5
409	0	-2	-23	28	1	3.1	11.9
410	-4	11	1	-16	-19	4.1	4.5
Group 2							
411	30	-18	-10	-32	77	4.3	3.3
412	5	-10	-46	-34	19	8.4	1.9
413	27	17	-15	25	12	2.9	0.6
414	34	42	7	0	-6	2.0	6.0
415	3	6	-2	-28	-4	2.8	3.2
416^b^	-	-	-	-	-	10.0	16.7
417^b^	-	-	-	-	-	10.0	0
418	25	21	-18	-66	5	4.0	3.9
419	30	20	-18	29	6	1.4	8.5

^a^Patient (401) received a bolus of 20 ml/kg/h instead of the 15mls/kg/h recommended by the World Health Organization. ^b^Patients 416 and 417 only had initial echocardiography as they died before post-rehydration measurements

### Cardiac function before and after fluid administration

Tables 4 and 3 present median (IQR) and percentage changes in the key cardiac and vascular performance indices and Additional file 6: Figure S3 presents box and whisker plots of echocardiography over time. On admission, median left ventricular fractional shortening (FS) in group 1 was within the normal range (28–44%) in 7/11 patients (64%), and improved modestly after fluid administration (Table 4). FS on admission was within the normal range in the same proportion of the children (6/9, 66%) in group 2 but changed little in the median FS after rehydration. In both groups the interquartile range of FS had reduced by 24 h compared to admission.

**Table 4:** Cardiac function parameter medians and interquartile ranges at different time points by study group

Parameter	Bolus + rehydration group (group 1)			Rehydration-only group (group 2)			*P* value*
	Pre fluid Median, (IQR)	Post fluid Median, (IQR)	24 h Median, (IQR)	Pre fluid Median, (IQR)	Post fluid Median, (IQR)	24 h Median, (IQR)	
FS (%)	28 (23, 31)	30 (24, 32)	29 (28, 31)	31 (23, 33)	31 (27, 35)	28 (25, 29)	0.89
IVCCI (%)	30 (23, 46)	38 (37, 39)	34 (23, 39)	38 (28, 44)	28 (24, 35)	23 (22, 26)	0.97
SVI (ml/min/m^2^)	30 (23, 45)	34 (27, 43)	33 (26, 37)	30 (24, 32)	32 (31, 41)	38 (32, 39)	0.81
CI (L/min/m^2^)	6.6 (4.0, 8.3)	6.3 (4.5, 7.5)	5.0 (4.6, 5.8)	6.9 (5.0, 8.5)	6.9 (4.9, 7.6)	6.2 (5.7, 7.0)	0.90
EDVI (ml/min/m^2^)	60 (48, 77)	62 (52, 76)	58 (44, 64)	50 (48, 59)	58 (51, 60)	74 (58, 76)	0.75
SVRI (ds/cm^5^/m^2^)	1349 (1039, 1797)	1275 (1189, 1610)	1422 (1294, 1556)	1265 (1155, 1969)	1105 (1071, 1655)	2102 (1883, 2113)	0.77
Tei index	0.28 (0.15, 0.40)	0.38 (0.10, 0.44)	0.27 (0.16, 0.32)	0.24 (0.17, 0.25)	0.15 (0.11, 0.32)	0.27 (0.25, 0.34)	0.73
Heart rate	106 (99, 113)	108 (102, 116)	111 (102, 129)	144 (109, 160)	130 (103, 170)	105 (103, 111)	0.19
GRS (%)	27 (12, 37)	24 (14, 36)	32 (13, 39)	21 (18, 29)	46 (31, 47)	32 (20, 34)	0.15
GCS (%)	-18 (-22, -15)	-19 (-25, -18)	-17 (-19, -14)	-19 (-25, -14)	-21 (-26, -18)	-21 (-21, -20)	0.81
GLS (%)	-23 (-24, -18)	-21 (-25, -15)	-22 (-25, -18)	-22 (-23, -19)	-20 (-23, -19)	-19 (-19, -17)	0.69

*FS* fractional shortening, *IVCCI* inferior vena cava collapsibility index, *SVI* stroke volume index, *CI* cardiac index, *EDVI* end-diastolic volume index, *SVRI* systemic vascular resistance index, *GRS* global radial strain, *GCS* global circumferential strain, *GLS* global longitudinal strain. * *P* value comparing post-fluid indices in group 1 vs. group 2

**Fig. 2:**
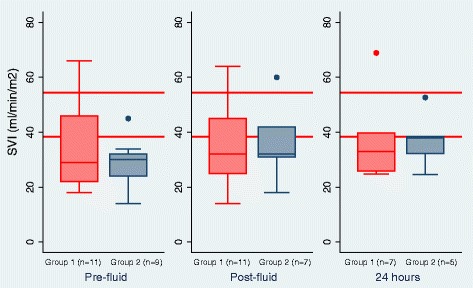
Stroke volume index (*SVI*) (ml/min/m^2^) by group. The red lines indicate published upper and lower limits of stroke volume index in children below 5 years [22]

As shown in Table 3 in group 1 the stroke volume index (SVI) increased by >10% after fluid bolus in 5/11 patients (suggesting fluid responsiveness) but decreased by >10% in two patients and remained unchanged (<5% change) after the fluid challenge in 4 patients. In group 2 the SVI after rehydration (no fluid bolus) was more homogenous, with no children having a decrease in SVI: in five patients there was >10% increase, and it remained unchanged in two patients. Two children died before post-rehydration measurements. Figure 2 provides a summary of the SVI by group over time.

**Fig. 3:**
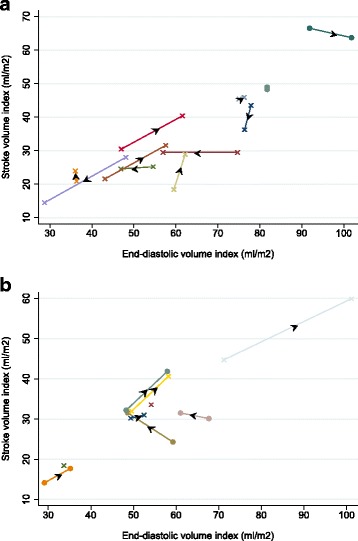
**a** Stroke volume index (*SVI*) in group1 plotted against end-diastolic volume index (*EDVI*) and (**b**) SVI in group 2 plotted against EDVI; pre-fluid, post-fluid *Arrows* indicate the direction of change in the SVI relative to the EDVI after fluid administration. *Solid circles* represent survivors. *Crosses* represent deaths

In order to evaluate possible echocardiographic signs of compromised heart function we related the fluid responsiveness (assessed by SVI) to a measure of compliance (assessed by the end-diastolic volume index (EDVI)). These are represented as Frank-Starling plots of SVI against EDVI in Fig. 3a and b. In four patients in group 1 the EDVI increased by >5%, which was associated with an increase in SVI (indicating fluid responsiveness) in three patients. However, in a fourth child (who survived) this was associated with a decrease in SVI (indicating non-fluid responsiveness). There was a very heterogeneous pattern of response in the other seven children, including either no change in EDVI after fluid administration or a decrease that was associated with an unchanged, increased or decreased SVI.

In group 2, EDVI increased in five patients after rehydration and this was associated with an increase in SVI in all patients, suggesting an “appropriate” myocardiac response to fluid therapy. SVI increased concomitant with a decrease in EDVI in two patients (one of these patients died). Two other patients died while undergoing initial rehydration prior to calculation of post-rehydration EDVI and SVI.

**Fig. 4:**
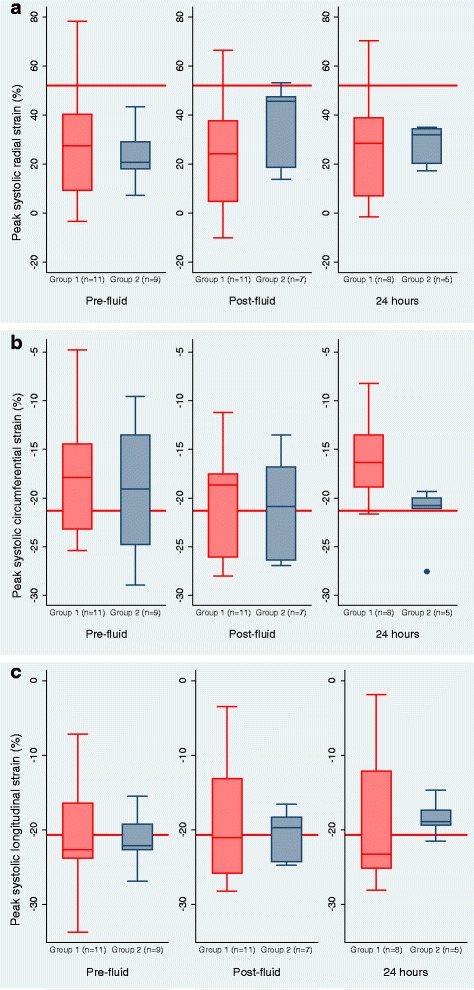
Global left-ventricle myocardial strain/deformation in (**a**) the radial and (**b**) the circumferential and (**c**) the longitudinal axes, by group. The red lines indicate published mean references of left ventricular cardiac strain in children from pooled meta-analysis [23]

Myocardial deformation/strain analysis was performed in the radial, circumferential and longitudinal axes (Fig. 4). Overall, the percentage global radial strain (GRS %) in these children with severe malnutrition and hypovolaemic shock was significantly lower than published references (mean GRS 26.2%, SE 4.1, 95% CI 17.4–33.8%; *p* < 0.05) [23]. Pericardial effusion was present in 11 children (55%), all of whom had kwashiorkor. Notably, the percentage global radial strain (GRS %) decreased after bolus fluid in group 1 but increased after rehydration in group 2. However, this difference between the groups after receiving fluid was not statistically significant (*F* = 0.33; *p* =0.15) (Table 4).

At baseline, the inferior vena cava collapsibility index (IVCCI), a surrogate marker of intravascular volume status [24–26], had a wide range of values across the two groups, but this narrowed after fluid administration, particularly in group 2. The interpretation of this is of questionable relevance, especially when many children had Kussmauls’ (acidotic) breathing. We noted that the median SVRI was generally high across the groups and despite initial reductions after fluid administration as expected, it rose again at 24 h and remained persistently high in the small number of survivors who were clinically well when reviewed at follow up on day 28.

With regard to cardiac biomarkers, the median brain natriuretic peptide (BNP) at admission was elevated (>300 pg/ml) in 6/10 patients (60%) in group 1 and 2/9 patients (22%) in group 2 (Additional file 7: Table S3). BNP remained elevated at 48 h in 4/7 patients (57%) in group 1 but not in any patients in group 2 (excluding two patients who died). Median troponin I was within the normal range in both groups at all the time points sampled. Electrophysiological recordings did not reveal any gross abnormalities or arrhythmias in any of the patients (Table 5).

**Table 5:** Electrocardiography (ECG) results (median and interquartile range)

ECG parameter	Group 1 Median (IQR)		Group 2 Median (IQR)		*P* value*
	Admission (n =11)	24 h (n = 8)	Admission (n = 9)	24 h (n = 5)	
P wave duration	78 (74, 145)	85 (73, 107)	88 (70, 160)	92 (92, 128)	0.53
PR interval	130 (111, 178)	127 (112, 215)	148 (102, 186)	130 (130, 182)	0.96
QRS duration	76 (66, 80)	75 (63, 78)	66 (64, 72)	70 (62, 74)	0.42
RR interval	546 (490, 579)	562 (513, 609)	460 (414, 540)	594 (540, 600)	0.96
PP interval	545 (490, 575)	565 (511, 610)	455 (415, 540)	590 (540, 600)	0.93
QT interval	318 (294, 378)	337 (293, 351)	344 (276, 360)	370 (276, 384)	0.59
QTc	461 (423, 509)	459 (437, 471)	473 (414, 509)	472 (422, 516)	0.64

*QTc* corrected QT-interval. * *P* value for comparison of post-fluid (24 h) indices in Group 1 vs. Group 2

### Mortality and adverse events

**Fig. 5:**
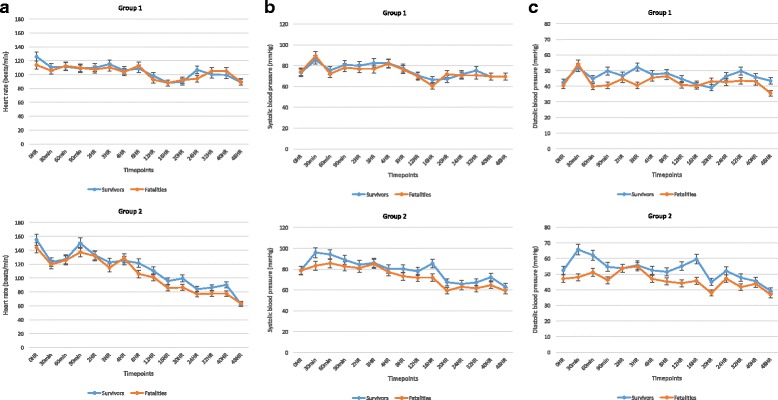
Median age-adjusted heart rate (**a**), systolic blood pressure (**b**) and diastolic blood pressure (**c**) by time and survival status

By 48 h, 5/11 patients (46%) and 3/9 patients (33%) in groups 1 and 2, respectively, had died. Overall 28-day mortality was higher in group 1 (9/11 patients (81.8%)) than in group 2 (5/9 patients (55.6%)) but the difference was not statistically significant (relative risk 0.902, 95% CI 0.313, 2.599; *p* = 0.8484) (Additional file 8: Figure S4). Median age-adjusted heart rate and blood pressure over time were similar in the survivors and fatalities with the exception of median diastolic blood pressure, which was lower in patients in group 2 who died before 4 h, and was consistent with uncorrected hypovolaemia in the early deaths (Fig. 5a-c).

In group 1 the median IVCCI worsened (increasing from 29% to 38%) post fluid administration in the non-survivors, while in the survivors the IVCCI decreased as expected (from a mean of 34% to 30%) post fluid resuscitation. In group 2, the median IVCCI reduced from 39% to 24% in the survivors, suggesting improved intravascular filling, and this observation was supported by a concomitant median increase in SVI from 30 to 32 ml/m^2^, whereas there was no clear trend in IVCCI in the non-survivors. As noted previously bradycardia at admission [8], present in 10% of patients (2/20) and low systolic blood pressure and persistent weak pulse after fluid administration in 55% of patients (11/20) were associated with early death, the majority occurring within 48 h of study admission.

We found no evidence of fluid overload adverse events in the study participants. This included children fulfilling the non-specific WHO criteria for CCF. That is, we found no evidence of increasing hepatomegaly or pulmonary oedema after fluid administration. None of the seven patients classified as having shock according to WHO guidelines at admission survived. Patients in whom BNP at 48 h remained persistently high (>300 pg/ml) died. HIV co-morbidity contributed to two deaths.

Fatal events can be classified into three time periods: early (0–24 h), intermediate (24–48 hour) and late (48 h to 15 days). In group 1 (receiving the WHO-recommended bolus, followed by maintenance) there were four early deaths (mainly within <8 h), which were all due to cardiovascular collapse secondary to hypovolaemia as a result of persistent high output (vomiting and stool). One child died at 48 h due to cardiovascular collapse (uncorrected acidosis/sepsis). The late deaths (n = 4) included two children who aspirated their nutritional feeds, and two children who died in the community. In group 2 (rehydration only) there were early deaths in three children who had cardiovascular collapse due to persistent hypovolaemic shock and another child had a fatal respiratory arrest at 20 h, which was associated with severe acidosis and hypoxaemia. Two late deaths occurred at day 4 (respiratory arrest associated with severe metabolic complications) and one child died in the community after 6 days. All fatal events were judged as unrelated to fluid challenges or the intravenous fluid regime, and largely were secondary to underlying co-morbidities (Additional file 9: Table S4 includes detailed clinical narratives and haemodynamic and echocardiographic findings).

## Discussion

We evaluated responses to two fluid rehydration regimes in high-risk severely malnourished children with hypovolaemic shock secondary to gastroenteritis, including 75% with the kwashiorkor phenotype. The intention of this study was to generate the relevant physiological evidence to support current guidelines. Overall, we found that neither baseline measurements, nor haemodynamic response to fluid resuscitation support the contention of compromised cardiac function indicative of heart failure, fluid overloading secondary to isotonic intravenous fluid resuscitation or rehydration, in African children with severe malnutrition.

Despite a high mortality rate, neither clinical nor echocardiographic data indicated evidence of volume overload leading to the adverse outcome. Instead, most deaths were related to underlying co-morbidities. The fluid bolus strategy led to diverse patterns of myocardial response, with both increased and decreased SVI, whereas with slower rehydration the responses were less heterogeneous, with most children having improvements in SVI. We found no significant differences overall between groups 1 and 2; however, comparison between the groups was limited by the small sample size in each group. However, these data form important pilot data for further investigation, given the existing equipoise in fluid volume, type and rate of administration for hypovolaemic shock resuscitation in severe malnutrition.

Severe malnutrition remains a common cause of hospital admission in Africa where the outcome remains poor, particularly in children with co-morbidities such as sepsis, gastroenteritis, pneumonia or HIV - conditions in which shock is a common complication. The WHO expert group recommends hypotonic solutions at lower doses for management of shock and for it to be given more slowly than in the general guidelines, principally to prevent development of heart failure or sodium overload [1]. The major concern in relation to the risk of these two complications largely pertains to the oedematous (kwashiorkor) phenotype. In this study, the majority of patients had the kwashiorkor phenotype, where we found no evidence to support these complications.

In reviewing the data on myocardial muscle mass and systolic and diastolic function for evidence to support current recommendations, we found these to be conflicting [27–30]. Most data from observational studies were collected at a single time point and the majority of the data were unconnected to the patient’s clinical condition or fluid management. Moreover, paediatric reference values for myocardial strain only reference non-malnourished children [23]. It was therefore not surprising that the radial strain was reduced, given the comparatively lower cardiac muscle mass in severe acute malnutrition (reflecting total body muscle mass), with pericardial effusion in more than half of the patients. Radial strain is a reflection of the outer sub-epicardial function of the myocardium and can often be reduced when there is pericardial pathologic change [31, 32]. The presence of pericardial effusion in 55% of our cohort may have been responsible for the observed reduction in radial strain. Strain has also been shown to be load-dependent and the presence of severe dehydration and very high SVRI at baseline, with significant reduction in preload, may be responsible for the lower baseline strain values across the board.

We were unable to find any relevant data validating definitions of paediatric congestive cardiac failure and myocardial compromise. First, we found that the WHO CCF criteria were non-specific, with substantial overlap with classical indicators of impaired circulatory or myocardial function related to shock and severe life-threatening illness (such as sepsis or severe metabolic acidosis).

Second, we found no evidence of gross myocardial dysfunction by either echocardiographic or by specific cardiac biomarkers. Troponin I is a volume-independent cardio-regulatory protein with high specificity and sensitivity for myocardial muscle damage [13, 14]. The low levels of troponin I in both study groups and the absence of supportive clinical, ECHO or ECG findings at all the time points do not support the hypothesis that the perturbations of cardiac function, reported by others [27, 28], are secondary to heart failure.

Third, in the majority of patients, myocardial and haemodynamic functional response to fluid administration led to initial improvement in the stroke volume index (SVI) and left ventricular fractional shortening (FS) and there were no adverse effects indicating fluid overload, which supported our concomitant clinical findings. The FS was within the normal range (28–44) at admission in 60% of children (12/20). Indeed, we found that median SVI increased by 16% in group 1 after fluid-bolus and by 5% in group 2 after fluid rehydration, while the left-ventricular internal diastolic diameter (LVIDd) increased by 3% in both groups after fluid administration, implying more optimal myocardial fibril compliance and improved contractility, and further demonstrated fluid responsiveness (FS) consistent with the upward slope of the Frank-Starling curve.

Finally, we observed high SVRI in both groups, which only slightly reduced after fluid administration but returning to supra-normal levels at 24 h. High SVRI has been previously observed in severely malnourished children, and this remained high independent of nutritional recovery from acute malnutrition (Brent et al.*,* CAPMAL study, unpublished data). This has also been previously described in adult survivors of childhood malnutrition [33, 34]. The possibility of local endothelial-mediated mechanisms contributing to the high SVRI requires further investigation to understand the evolution and management of hypovolaemic shock. BNP is a volume-dependent cardiac hormone released in the ventricles in response to volume expansion and pressure loading [35–38]. It has diuretic, natriuretic and vasodilator properties [39] and has been used to predict outcome in paediatric heart failure [12]; nevertheless, it has also been shown to be high in other conditions (sepsis, shock and pneumonia) so it is vital that absolute values are interpreted alongside clinical status [40–42]. The persistent elevation of BNP alongside a high SVRI requires further investigation. However, one of limitations of this study was the lack of central venous pressure (CVP) validation of the SVRI index.

We were not able to elucidate the potential mechanism for the poor outcome in this high-risk group of children with severe malnutrition. Nonetheless, our clinical data indicate that poor outcomes were unrelated to fluid challenges. Electrolyte and metabolic derangements in severe malnutrition have also been shown to carry a high risk of mortality [39, 40] and this was a common complication. Contrary to previous reports of sudden and unexpected deaths mainly seen in oedematous severely malnourished children, which attribute the high mortality rates to cardiac arrhythmias or congestive cardiac failure after fluid administration [43, 44], we did not find evidence to support either the development of cardiac failure or lethal arrhythmia.

## Conclusion

In a physiological study examining clinical, haemodynamic and myocardial responses to intravenous fluid expansion in the management of a high-risk group of severely malnourished children with hypovolaemic shock, we found no evidence at baseline or after fluid challenge of the putative complication of cardiac failure. This view is widely held as a major reason to withhold adequate intravenous volume replacement in children with severe malnutrition. The outcome, nevertheless, remained poor, but appeared unrelated to fluid overload. Further research is required to investigate whether more liberal fluid rehydration strategies may be beneficial to the immediate outcome, linked to relevant physiological studies to understand the pathophysiologic mechanisms underlying the high rate of late mortality in children with severe malnutrition complicated by gastroenteritis.

## Additional files

Additional file 1: Box 1 Echocardiographic cardiac measurement formulae. (PDF 50 kb)

Additional file 2: Table S1. a Clinical features (median, interquartile range and standard deviation) and prevalence (*n*; %) of abnormal signs at different time points in group 1 (bolus + rehydration). **b** Clinical features (median, interquartile range and standard deviation) and prevalence (*n*; %) of abnormal signs at different time points in group two (rehydration-only). (ZIP 55 kb)

Additional file 3: Figure S1. By study group over time median heart rate (a) non-adjusted and (b) age-adjusted; median systolic blood pressure (c) nonadjusted and (d) age-adjusted; median diastolic blood pressure (e) nonadjusted and (f) age-adjusted. (PDF 449 kb)

Additional file 4: Table S2 Blood measurements (median and inter-quartile range) at different time points by study group. (PDF 37 kb)

Additional file 5: Figure S2 Blood test parameters. **a** Lactate. **b** Haemoglobin. **c** Potassium. **d** Sodium. **e** White blood cell count. **f** Creatinine. (PDF 59 kb)

Additional file 6: Figure S3 Cardiac haemodynamic parameters. **a** Fractional shortening. **b** Cardiac index. **c** Left ventricle end-diastolic volume index. **d** Systemic vascular resistance index. **e** Inferior vena cava collapsibility index. (PDF 19 kb)

Additional file 7: Table S3. Cardiac bio-markers (medians, interquartile ranges and standard deviation) at admission and 48 h, by study group. (DOC 74 kb)

Additional file 8: Figure S4. Kaplan-Meier survival estimates at 48 h (**a**) and day 28 (b), by study group. (DOCX 35 kb)

Additional file 9: Table S4. Detailed narratives of the severe adverse events (SAEs). (PDF 25 kb)

## Abbreviations

AFRIM: Appropriate Fluid Resuscitation In Malnutrition
AVET: Aortic valve ejection time
BNP: Brain natriuretic peptide
BSA: Body surface area
BT: Blood transfusion
CAPMAL: Cardiac physiology assessment in severe malnutrition study
CCF: Congestive cardiac failure
CI: Cardiac index
CO: Cardiac output
CVP: Central venous pressure
ECG: Electrocardiogram
ECHO: Echocardiogram
EDD: End-diastolic diameter
EDV: End-diastolic volume
EDVI: End-diastolic volume index
EPLS: European Paediatric Life Support
ERC: Ethical Review Committee
ESD: End-systolic diameter
ESV: End-systolic volume
FEAST: Fluid Expansion as A Supportive Therapy trial
FIM: Fluids In Malnutrition trial
FR: Fluid responsiveness
FS: Fractional shortening
Hb: Haemoglobin
HIV: Human-immunodeficiency virus
HR: Heart rate
HSD/5D: Half-strength Darrow’s solution/5% dextrose
ICT: Isovolumic contraction time
IMCI: Integrated management of childhood illness
IQR: Inter-quartile range
IRC: Institutional Review Committee
IVC: Inferior vena cava
IVCCI: Inferior vena cava collapsibility index
IVT: Isovolumic relaxation time
JVP: Jugular Venous Pressure
KEMRI: Kenya Medical Research Institute
KWTRP: KEMRI-Wellcome Trust Research Programme
LV: Left ventricle
LVIDd: Left ventricle internal diastolic diameter
MCO: Mitral valve closure to opening time
MRRH: Mbale Regional Referral Hospital
MUAC: Mid-upper arm circumference
PLAX: Para-sternal long axis
PSAX: Para-sternal short axis
ReSoMal: Rehydration solution for malnutrition
RL: Ringer’s lactate
SV: Stroke volume
SVI: Stroke volume index
SVR: Systemic vascular resistance
SVRI: Systemic vascular resistance index
VTI: Velocity time integral
WHO: World Health Organization
WHZ: Weight-for-height Z score

## References

[1] WHO. Pocket book of hospital care for children 2nd edition. Guidelines for the management of common childhood illnesses. Geneva: WHO; 2013. 24006557

[2] Akech SO, Karisa J, Nakamya P, Boga M, Maitland K (2010). Phase II trial of isotonic fluid resuscitation in Kenyan children with severe malnutrition and hypovolaemia. BMC Pediatr.

[3] Brewster DR (2006). Critical appraisal of the management of severe malnutrition: 1. Epidemiology and treatment guidelines. J Paediatr Child Health.

[4] Alam NH, Islam S, Sattar S, Monira S, Desjeux JF (2009). Safety of rapid intravenous rehydration and comparative efficacy of 3 oral rehydration solutions in the treatment of severely malnourished children with dehydrating cholera. J Pediatr Gastroenterol Nutr.

[5] Ahmed T, Ali M, Ullah MM, Choudhury IA, Haque ME, Salam MA, Rabbani GH, Suskind RM, Fuchs GJ (1999). Mortality in severely malnourished children with diarrhoea and use of a standardised management protocol. Lancet.

[6] Maitland K, Kiguli S, Opoka RO, Engoru C, Olupot-Olupot P, Akech SO, Nyeko R, Mtove G, Reyburn H, Lang T (2011). Mortality after fluid bolus in African children with severe infection. N Engl J Med.

[7] Talbert A, Thuo N, Karisa J, Chesaro C, Ohuma E, Ignas J, Berkley JA, Toromo C, Atkinson S, Maitland K (2012). Diarrhoea complicating severe acute malnutrition in Kenyan children: a prospective descriptive study of risk factors and outcome. PLoS One.

[8] Maitland K, Berkley JA, Shebbe M, Peshu N, English M, Newton CR (2006). Children with severe malnutrition: can those at highest risk of death be identified with the WHO protocol?. PLoS Med.

[9] Maitland K, Molyneux S, Boga M, Kiguli S, Lang T (2011). Use of deferred consent for severely ill children in a multi-centre phase III trial. Trials.

[10] Maitland K, George EC, Evans JA, Kiguli S, Olupot-Olupot P, Akech SO, Opoka RO, Engoru C, Nyeko R, Mtove G (2013). Exploring mechanisms of excess mortality with early fluid resuscitation: insights from the FEAST trial. BMC Med.

[11] Koch A, Singer H (2003). Normal values of B type natriuretic peptide in infants, children, and adolescents. Heart.

[12] Auerbach SR, Richmond ME, Lamour JM, Blume ED, Addonizio LJ, Shaddy RE, Mahony L, Pahl E, Hsu DT (2010). BNP levels predict outcome in pediatric heart failure patients: post hoc analysis of the Pediatric Carvedilol Trial. Circ Heart Fail.

[13] Towbin JA, Gajarski RJ (1997). Cardiac troponin I: a new diagnostic gold standard of cardiac injury in children?. J Pediatr.

[14] Soldin SJ, Murthy JN, Agarwalla PK, Ojeifo O, Chea J (1999). Pediatric reference ranges for creatine kinase, CKMB, Troponin I, iron, and cortisol. Clin Biochem.

[15] Price JF, Thomas AK, Grenier M, Eidem BW, O’Brian Smith E, Denfield SW, Towbin JA, Dreyer WJ (2006). B-type natriuretic peptide predicts adverse cardiovascular events in pediatric outpatients with chronic left ventricular systolic dysfunction. Circulation.

[16] Lopez L, Colan SD, Frommelt PC, Ensing GJ, Kendall K, Younoszai AK, Lai WW, Geva T (2010). Recommendations for quantification methods during the performance of a pediatric echocardiogram: a report from the Pediatric Measurements Writing Group of the American Society of Echocardiography Pediatric and Congenital Heart Disease Council. J Am Soc Echocardiogr.

[17] Bazett HC (1920). An analysis of the time-relations of electrocardiograms. Heart-J Stud Circ.

[18] Tutar HE, Ocal B, Imamoglu A, Atalay S (1998). Dispersion of QT and QTc interval in healthy children, and effects of sinus arrhythmia on QT dispersion. Heart.

[19] Faddan NH, Sayh KI, Shams H, Badrawy H (2010). Myocardial dysfunction in malnourished children. Ann Pediatr Cardiol.

[20] Marcelino P, Fernandes AP, Marum S, Ribeiro JP (2002). Non-invasive evaluation of central venous pressure by echocardiography. Rev Port Cardiol.

[21] Groeneveld AB, Nauta JJ, Thijs LG (1988). Peripheral vascular resistance in septic shock: its relation to outcome. Intensive Care Med.

[22] Cattermole GN, Leung PY, Mak PS, Chan SS, Graham CA, Rainer TH (2010). The normal ranges of cardiovascular parameters in children measured using the Ultrasonic Cardiac Output Monitor. Crit Care Med.

[23] Jashari H, Rydberg A, Ibrahimi P, Bajraktari G, Kryeziu L, Jashari F, Henein MY (2015). Normal ranges of left ventricular strain in children: a meta-analysis. Cardiovasc Ultrasound.

[24] Marik PE, Baram M, Vahid B (2008). Does central venous pressure predict fluid responsiveness? A systematic review of the literature and the tale of seven mares. Chest.

[25] Marik PE, Cavallazzi R (2013). Does the central venous pressure predict fluid responsiveness? An updated meta-analysis and a plea for some common sense. Crit Care Med.

[26] Marik PE, Lemson J (2014). Fluid responsiveness: an evolution of our understanding. Br J Anaesth.

[27] Viart P (1978). Hemodynamic findings during treatment of protein-calorie malnutrition. Am J Clin Nutr.

[28] Phornphatkul C, Pongprot Y, Suskind R, George V, Fuchs G (1994). Cardiac function in malnourished children. Clin Pediatr.

[29] Kothari SS, Patel TM, Shetalwad AN, Patel TK (1992). Left ventricular mass and function in children with severe protein energy malnutrition. Int J Cardiol.

[30] Ocal B, Unal S, Zorlu P, Tezic HT, Oguz D (2001). Echocardiographic evaluation of cardiac functions and left ventricular mass in children with malnutrition. J Paediatr Child Health.

[31] Leitman M, Bachner-Hinenzon N, Adam D, Fuchs T, Theodorovich N, Peleg E, Krakover R, Moravsky G, Uriel N, Vered Z (2011). Speckle tracking imaging in acute inflammatory pericardial diseases. Echocardiography.

[32] Sengupta PP, Tajik AJ, Chandrasekaran K, Khandheria BK (2008). Twist mechanics of the left ventricle: principles and application. J Am Coll Cardiol Img.

[33] Emery PW. Metabolic changes in malnutrition. Eye. 2005;19(10):1029–34. 10.1038/sj.eye.6701959 16304580

[34] Bai Z, Zhu X, Li M, Hua J, Li Y, Pan J, Wang J, Li Y. Effectiveness of predicting in-hospital mortality in critically ill children by assessing blood lactate levels at admission. BMC Pediatr. 2014;14:83. 10.1186/1471-2431-14-83 PMC3976355 24673817

[35] Yandle TG. Biochemistry of natriuretic peptides. J Intern Med. 1994;235(6):561–76. 10.1111/j.1365-2796.1994.tb01263.x 8207362

[36] Nakagawa O, Ogawa Y, Itoh H, Suga S, Komatsu Y, Kishimoto I, Nishino K, Yoshimasa T, Nakao K. Rapid transcriptional activation and early mRNA turnover of brain natriuretic peptide in cardiocyte hypertrophy. Evidence for brain natriuretic peptide as an “emergency” cardiac hormone against ventricular overload. J Clin Invest. 1995;96(3):1280–7. 10.1172/JCI118162 PMC185749 7657802

[37] Maeda K, Tsutamoto T, Wada A, Hisanaga T, Kinoshita M. Plasma brain natriuretic peptide as a biochemical marker of high left ventricular end-diastolic pressure in patients with symptomatic left ventricular dysfunction. Am Heart J. 1998;135(5 Pt 1):825–32. 10.1016/s0002-8703(98)70041-9 9588412

[38] Yoshimura M, Yasue H, Okumura K, Ogawa H, Jougasaki M, Mukoyama M, Nakao K, Imura H. Different secretion patterns of atrial natriuretic peptide and brain natriuretic peptide in patients with congestive heart failure. Circulation. 1993;87(2):464–9. 10.1161/01.cir.87.2.464 8425293

[39] Levin ER, Gardner DG, Samson WK. Natriuretic peptides. N Engl J Med. 1998;339(5):321–8. 10.1056/NEJM199807303390507 9682046

[40] McLean AS, Huang SJ, Hyams S, Poh G, Nalos M, Pandit R, Balik M, Tang B, Seppelt I. Prognostic values of B-type natriuretic peptide in severe sepsis and septic shock. Crit Care Med. 2007;35(4):1019–26. 10.1097/01.CCM.0000259469.24364.31 17334249

[41] Pirracchio R, Deye N, Lukaszewicz AC, Mebazaa A, Cholley B, Mateo J, Megarbane B, Launay JM, Peynet J, Baud F, et al. Impaired plasma B-type natriuretic peptide clearance in human septic shock. Crit Care Med. 2008;36(9):2542–6. 10.1097/CCM.0b013e318183f067 18679125

[42] Nikolaou NI, Goritsas C, Dede M, Paissios NP, Papavasileiou M, Rombola AT, Ferti A. Brain natriuretic peptide increases in septic patients without severe sepsis or shock. Eur J Intern Med. 2007;18(7):535–41. 10.1016/j.ejim.2007.01.006 17967335

[43] Dramaix M, Brasseur D, Donnen P, Bawhere P, Porignon D, Tonglet R, Hennart P. Prognostic indices for mortality of hospitalized children in central Africa. Am J Epidemiol. 1996;143(12):1235–43. 10.1093/oxfordjournals.aje.a008711 8651222

[44] Bachou H, Tumwine JK, Mwadime RK, Tylleskar T. Risk factors in hospital deaths in severely malnourished children in Kampala. Uganda BMC Pediatr. 2006;6:7. 10.1186/1471-2431-6-7 PMC1472687 16542415

[45] Tei C, Ling LH, Hodge DO, Bailey KR, Oh JK, Rodeheffer RJ, Tajik AJ, Seward JB (1995). New index of combined systolic and diastolic myocardial performance: a simple and reproducible measure of cardiac function–a study in normals and dilated cardiomyopathy. J Cardiol.

[46] Tei C (1995). New non-invasive index for combined systolic and diastolic ventricular function. J Cardiol.

[47] Karatzis EN, Giannakopoulou AT, Papadakis JE, Karazachos AV, Nearchou NS (2009). Myocardial performance index (Tei index): evaluating its application to myocardial infarction. Hellenic J Cardiol.

