# Supplementary material for: Myocardial and haemodynamic responses to two fluid regimens in African children with severe malnutrition and hypovolaemic shock (AFRIM study)
Source: Crit Care. 2017 May 3;21:103. doi: 10.1186/s13054-017-1679-0 (PMC5415747; doi:10.1186/s13054-017-1679-0)
Supplement: Supplementary file 1 — Box 1 Echocardiographic cardiac measurement formulae. (PDF 50 kb) [file 13054_2017_1679_MOESM1_ESM.pdf]

Supplemental box 1: Echocardiographic cardiac measurement formulae

The modified Simpson's algorithm using a combination of short-axis and long-axis views was used to calculate LV diastolic and systolic volumes [1], based on anticipated changes in LV mass and shape in malnutrition. The area-length formula was used to calculate the LV volumes [2-4]: Volume = 5/6 X parasternal short-axis basal area X Apical LV length.

The stroke volume (SV) was derived from the difference between the end-diastolic volume (EDV) and the end-systolic volume (ESV). Fractional shortening (FS) was measured as the percentage change in LV diameter during systole and diastole using the formula [4]: FS = [(LVEDD – LVESD)/LVEDD] X 100

Cardiac output (CO) was obtained by multiplication of the stroke volume (SV) with the heart rate (HR) recorded on the ECG. The systemic vascular resistance (SVR) was calculated using Darcy's formula that relates pressure, flow and resistance by dividing the mean blood pressure by the aortic velocity time integral (VTI) flow. Mean arterial pressure was derived from measured systolic and diastolic blood pressure values and height and weight data were directly entered at the beginning of the echo assessment. The measurements of SV, EDV, CO and SVR were all indexed to the patients' body surface area (BSA) derived in the *Vivid.i* echocardiogram using the configured Haycock formula [5]: BSA (m^2) = 0.024265 x weight (kg) $^{0.5378}$ x height (cm) $^{0.3964}$

The maximum and minimum inferior vena cava (IVC) diameter was measured 2cm from the right atrium in the sub-costal view and the IVC collapsibility index (IVCCI) calculated as a percentage using the formula [6]: IVCCI = $[(IVCd_{max} - IVCd_{min})/IVCd_{max}]$ X 100

Myocardial deformation was assessed by speckle tracking and quantification of the mean global radial, circumferential and longitudinal strain in the parasternal short axis and apical views with 2 dimensional speckle tracking [7, 8] and compared with published reference values [9]. Global strain analysis was quantified using vendor-specific offline analysis software EchoPAC BT13 (GE, Milwaukee). GLS was derived from the weighted average strain of all 17 segments from the images of 3 apical views (apical long-axis, 4- and 2-chamber). GCS and GRS were derived by taking the average strain values of the 6 segments in the mid LV short axis view. Care was taken to ensure all images were acquired within the recommended frame rate of 50-80 frames per second (FPS).

Myocardial performance was defined based on the Tei index, which was calculated by dividing the sum of the isovolumic contraction time (IVCT) and isovolumic relaxation time (IVRT) by the aortic valve ejection time (AVET) using the formula [10-13]: Tei index = [(IVCT + IVRT)/AVET.

Whereby, the sum of IVCT and IVRT is equivalent to the difference between the mitral valve closure to opening (MCO) time and the AVET (i.e. [IVCT + IVRT] = [MCO – AVET])

References:

1. Mercier JC, DiSessa TG, Jarmakani JM, Nakanishi T, Hiraishi S, Isabel-Jones J, Friedman WF: **Two-dimensional echocardiographic assessment of left ventricular volumes and ejection fraction in children**. *Circulation* 1982, **65**(5):962-969.
2. Wyatt HL, Heng MK, Meerbaum S, Gueret P, Hestenes J, Dula E, Corday E: **Cross-sectional echocardiography. II. Analysis of mathematic models for quantifying volume of the formalin-fixed left ventricle**. *Circulation* 1980, **61**(6):1119-1125.
3. Wyatt HL, Meerbaum S, Heng MK, Gueret P, Corday E: **Cross-sectional echocardiography. III. Analysis of mathematic models for quantifying volume of symmetric and asymmetric left ventricles**. *Am Heart J* 1980, **100**(6 Pt 1):821-828.
4. Lopez L, Colan SD, Frommelt PC, Ensing GJ, Kendall K, Younoszai AK, Lai WW, Geva T: **Recommendations for quantification methods during the performance of a pediatric echocardiogram: a report from the Pediatric Measurements Writing Group of the American Society of Echocardiography Pediatric and Congenital Heart Disease Council**. *J Am Soc Echocardiogr* 2010, **23**(5):465-495; quiz 576-467.
5. Haycock GB, Schwartz GJ, Wisotsky DH: **Geometric method for measuring body surface area: a height-weight formula validated in infants, children, and adults**. *J Pediatr* 1978, **93**(1):62-66.
6. Kutty S, Li L, Hasan R, Peng Q, Rangamani S, Danford DA: **Systemic venous diameters, collapsibility indices, and right atrial measurements in normal pediatric subjects**. *J Am Soc Echocardiogr* 2014, **27**(2):155-162.
7. Gorcsan J, 3rd, Tanaka H: **Echocardiographic assessment of myocardial strain**. *J Am Coll Cardiol* 2011, **58**(14):1401-1413.
8. Lang RM, Badano LP, Mor-Avi V, Afilalo J, Armstrong A, Ernande L, Flachskampf FA, Foster E, Goldstein SA, Kuznetsova T *et al*: **Recommendations for cardiac chamber quantification by echocardiography in adults: an update from the American Society of Echocardiography and the European Association of Cardiovascular Imaging**. *J Am Soc Echocardiogr* 2015, **28**(1):1-39 e14.
9. Jashari H, Rydberg A, Ibrahimi P, Bajraktari G, Kryeziu L, Jashari F, Henein MY: **Normal ranges of left ventricular strain in children: a meta-analysis**. *Cardiovascular ultrasound* 2015, **13**:37.
10. Tei C, Ling LH, Hodge DO, Bailey KR, Oh JK, Rodeheffer RJ, Tajik AJ, Seward JB: **New index of combined systolic and diastolic myocardial performance: a simple and reproducible measure of cardiac function--a study in normals and dilated cardiomyopathy**. *J Cardiol* 1995, **26**(6):357-366.
11. Tei C: **New non-invasive index for combined systolic and diastolic ventricular function**. *J Cardiol* 1995, **26**(2):135-136.
12. Karatzis EN, Giannakopoulou AT, Papadakis JE, Karazachos AV, Nearchou NS: **Myocardial performance index (Tei index): evaluating its application to myocardial infarction**. *Hellenic journal of cardiology : HJC = Hellenike kardiologike epitheorese* 2009, **50**(1):60-65.
13. Karaye KM: **Relationship between Tei Index and left ventricular geometric patterns in a hypertensive population: a cross-sectional study**. *Cardiovasc Ultrasound* 2011, **9**:21.
