# Supplementary material for: Myocardial and haemodynamic responses to two fluid regimens in African children with severe malnutrition and hypovolaemic shock (AFRIM study)
Source: Crit Care. 2017 May 3;21:103. doi: 10.1186/s13054-017-1679-0 (PMC5415747; doi:10.1186/s13054-017-1679-0)
Supplement: Supplementary file 2 — a Clinical features (median, interquartile range and standard deviation) and prevalence (n; %) of abnormal signs at different time points in group 1 (bolus + rehydration). b Clinical features (median, interquartile range and standard deviation) and prevalence (n; %) of abnormal signs at different time points in group two (rehydration-only). (ZIP 55 kb) [file 13054_2017_1679_MOESM2_ESM.zip › AFRIM supplemental table 1a.pdf]

Supplemental table 1 (a): Clinical features (median, interquartile range and standard deviation) and prevalence (n; %) of abnormal signs at different time-points for Group one (bolus + rehydration)

	**Pre-fluid (n=11)**				**Post-fluid (n=10)**				**48-hours (n=7)**				**Day 28, follow-up (n=2)**		
	Med	IQR	SD		Med	IQR	SD		Med	IQR	SD		Med	IQR	SD
**Axillary Temp (^{0}C)**	**35.1**	**(33.9, 36.0)**	**1.23**		**36.0**	**(35.6, 36.1)**	**1.02**		**36.6**	**(36.1, 36.8)**	**0.55**		**36.5**	**(36.2, 36.8)**	**0.85**
Hypothermia	5	(45.5%)			2	(20%)			0	(0%)			0	(0%)	
Fever	0	(0%)			0	(0%)			0	(0%)			0	(0%)	
Temperature gradient*	11	(100%)			7	(70%)			2	(28.6%)			0	(0%)	
**Respiratory rate (breaths/min)**	**37**	**(26, 42)**	**12.92**		**32**	**(29, 37)**	**13.33**		**44**	**(39, 49)**	**6.53**		**28**	**(23, 33)**	**14.14**
Tachypnoea	4	(36.4%)			2	(20%)			4	(57.1%)			0	(0%)	
Chest indrawing	11	(100%)			9	(90%)			4	(57.1%)			0	(0%)	
Deep breathing	9	(82%)			6	(60%)			2	(28.6%)			0	(0%)	
**Oxygen saturation**	**96**	**(83, 99)**	**13.45**		**98**	**(96, 99)**	**6.33**		**98**	**(98, 99)**	**3.24**		**95**	**(95, 95)**	**0**
Hypoxia (<90%)	5	(45.5%)			0	(0%)			1	(14.3%)			0	(0%)	
**Pulse (beats/min)**	**106**	**(99, 113)**	**20.37**		**112**	**(103, 118)**	**19.14**		**115**	**(111, 121)**	**8.42**		**115**	**(112, 117)**	**6.36**
Tachycardia	1	(9.1%)			1	(10%)			0	(0%)			0	(0%)	
Bradycardia	1	(9.1%)			0	(0%)			0	(0%)			0	(0%)	
Weak pulse	7	(63.6%)			6	(60%)			0	(0%)			0	(0%)	
**Systolic blood pressure (mmHg)**	**77**	**(72, 81)**	**10.55**		**77**	**(77, 89)**	**13.61**		**81**	**(77, 96)**	**20.26**		**111**	**(104, 117)**	**17.68**
Hypotension	3	(27.3%)			1	(10%)			0	(0%)			0	(0%)	
**Capillary refill time, CRT (seconds)**	**2**	**(2, 3)**	**0.93**		**2**	**(1, 2)**	**1.18**		**1**	**(1, 1)**	**0**		**3**	**(2, 3)**	**0.71**
Prolonged CRT	5	(45.5%)			2	(20%)			0	(0%)			1	(50%)	

The medians, interquartile range (IQR) and standard deviation (SD) are presented for each clinical feature, as well as proportions outside the normal reference ranges
*Temperature gradient is difference in temperature between peripheral extremities and central body mass; usually assessed on the limbs from toes ascending towards the head; hypothermia (axillary temperature < 35.0^{0}C); fever (axillary temperature > 37.5^{0}C); tachypnoea (respiratory rate >40 breaths/min); hypoxia (oxygen saturation <90%); tachycardia (heart rate >160/min in a child <12 months; >120/min in a child aged 12 months to 5 years); bradycardia (heart rate <80 beats/min); prolonged capillary refill time $\geq$3seconds.
