# Supplementary material for: Myocardial and haemodynamic responses to two fluid regimens in African children with severe malnutrition and hypovolaemic shock (AFRIM study)
Source: Crit Care. 2017 May 3;21:103. doi: 10.1186/s13054-017-1679-0 (PMC5415747; doi:10.1186/s13054-017-1679-0)
Supplement: Supplementary file 2 — a Clinical features (median, interquartile range and standard deviation) and prevalence (n; %) of abnormal signs at different time points in group 1 (bolus + rehydration). b Clinical features (median, interquartile range and standard deviation) and prevalence (n; %) of abnormal signs at different time points in group two (rehydration-only). (ZIP 55 kb) [file 13054_2017_1679_MOESM2_ESM.zip › AFRIM supplemental table 1b.pdf]

Supplemental table 1 (b): Clinical features (median, interquartile range and standard deviation) and prevalence (n; %) of abnormal signs at different time-points for Group two (rehydration-only)

	Pre-fluid (n=9)				Post-fluid (n=8)				48-hours (n=5)				Day 28, follow-up (n=2)		
	Med	IQR	SD		Med	IQR	SD		Med	IQR	SD		Med	IQR	SD
**Axillary Temp (^{0}C)**	35.9	(35.2, 36.6)	1.08		37.0	(36.4, 37.6)	1.22		36.0	(35.7, 36.5)	0.61		35.5	(35.4, 35.5)	0.07
Hypothermia	2	(22.2%)			1	(12.5%)			0	(0%)			0	(0%)	
Fever	0	(0%)			2	(25%)			0	(0%)			0	(0%)	
Temperature gradient*	9	(100%)			6	(75%)			2	(40%)			0	(0%)	
**Respiratory rate (breaths/min)**	46	(41, 53)	13.25		46	(34, 48)	11.09		36	(35, 38)	10.01		24	(22, 25)	4.95
Tachypnoea	6	(66.7%)			5	(62.5%)			1	(20%)			0	(0%)	
Chest indrawing	9	(100%)			8	(100%)			1	(20%)			0	(0%)	
Deep breathing	4	(44.4%)			2	(25%)			1	(20%)			0	(0%)	
**Oxygen saturation**	91	(82, 98)	22.09		98	(96, 98)	2.67		98	(96, 98)	4.49		97	(96, 97)	2.12
Hypoxia	5	(55.6%)			1	(12.5%)			1	(20%)			0	(0%)	
**Pulse (beats/min)**	144	(109, 160)	35.85		131	(103, 170)	38.95		108	(105, 112)	9.29		136	(119, 152)	45.96
Tachycardia	4	(44.4%)			4	(50%)			0	(0%)			1	(50%)	
Bradycardia	1	(11.1%)			0	(0%)			0	(0%)			0	(0%)	
Weak pulse	6	(66.7%)			5	(62.5%)			0	(0%)			0	(0%)	
**Systolic blood pressure (mmHg)**	81	(77, 82)	20.39		82	(78, 93)	13.83		91	(80, 92)	8.17		93	(88, 98)	14.14
Hypotension	2	(22.2%)			1	(12.5%)			0	(0%)			0	(0%)	
**Capillary refill time, CRT (seconds)**	2	(2, 4)	1.17		2	(1, 2)	1.13		1	(1, 2)	0.55		2	(1, 2)	0.71
Prolonged CRT	4	(44.4%)			2	(25%)			0	(0%)			0	(0%)	

The medians, interquartile range (IQR) and standard deviation (SD) are presented for each clinical feature, as well as proportions outside the normal reference ranges
*Temperature gradient is difference in temperature between peripheral extremities and central body mass; usually assessed on the limbs from toes ascending towards the head; hypothermia (axillary temperature < 35.0^{0}C); fever (axillary temperature > 37.5^{0}C); tachypnoea (respiratory rate >40 breaths/min); hypoxia (oxygen saturation <90%); tachycardia (heart rate >160/min in a child <12 months; >120/min in a child aged 12 months to 5 years); bradycardia (heart rate <80 beats/min); prolonged capillary refill time $\geq$3seconds.
