# Supplementary figures and images for: Myocardial and haemodynamic responses to two fluid regimens in African children with severe malnutrition and hypovolaemic shock (AFRIM study)
Source: Crit Care. 2017 May 3;21:103. doi: 10.1186/s13054-017-1679-0 (PMC5415747; doi:10.1186/s13054-017-1679-0)

1(a)

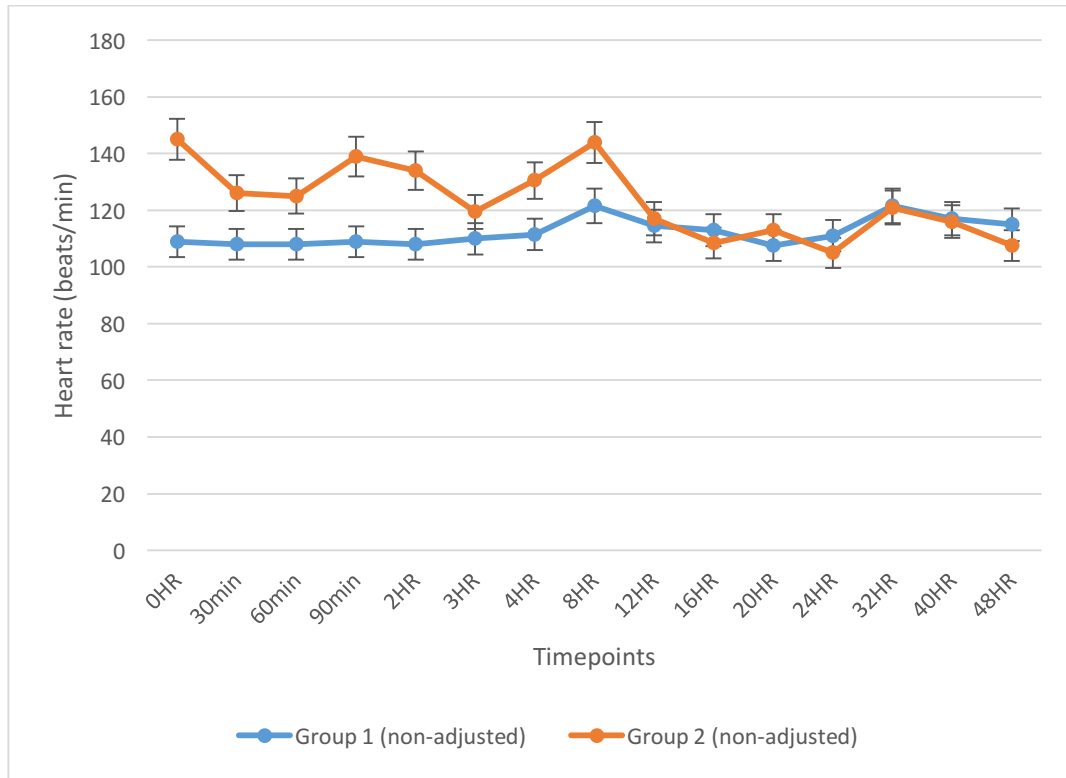

1(b)

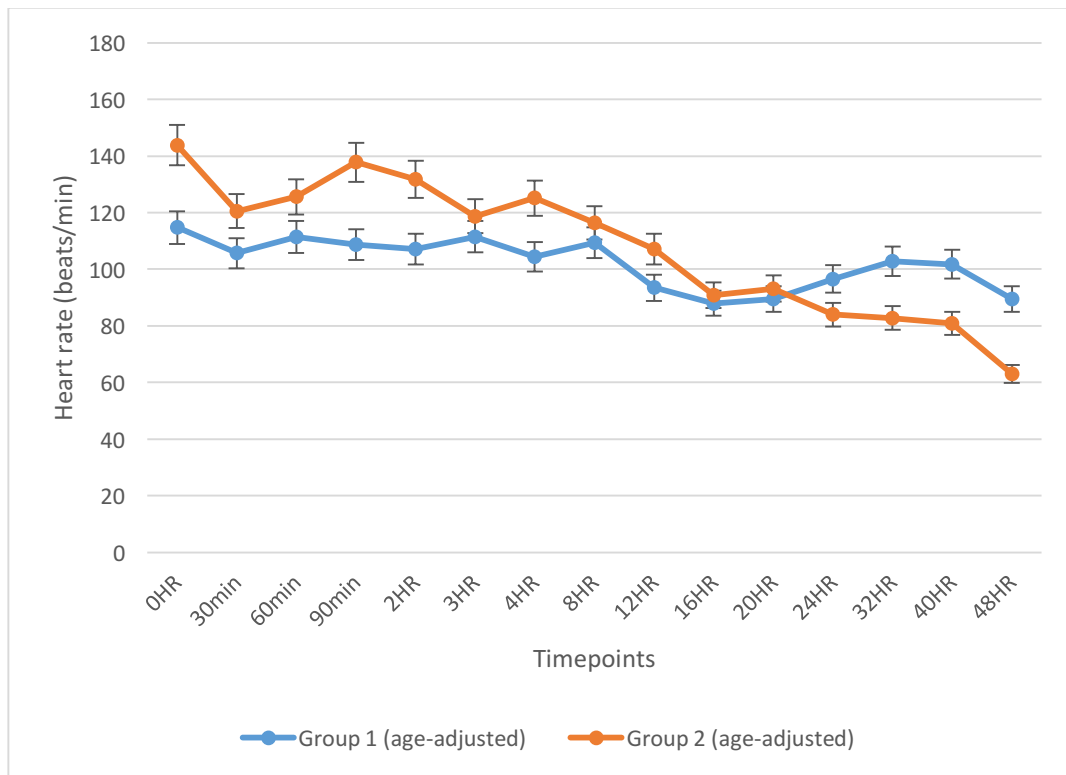

1(c)

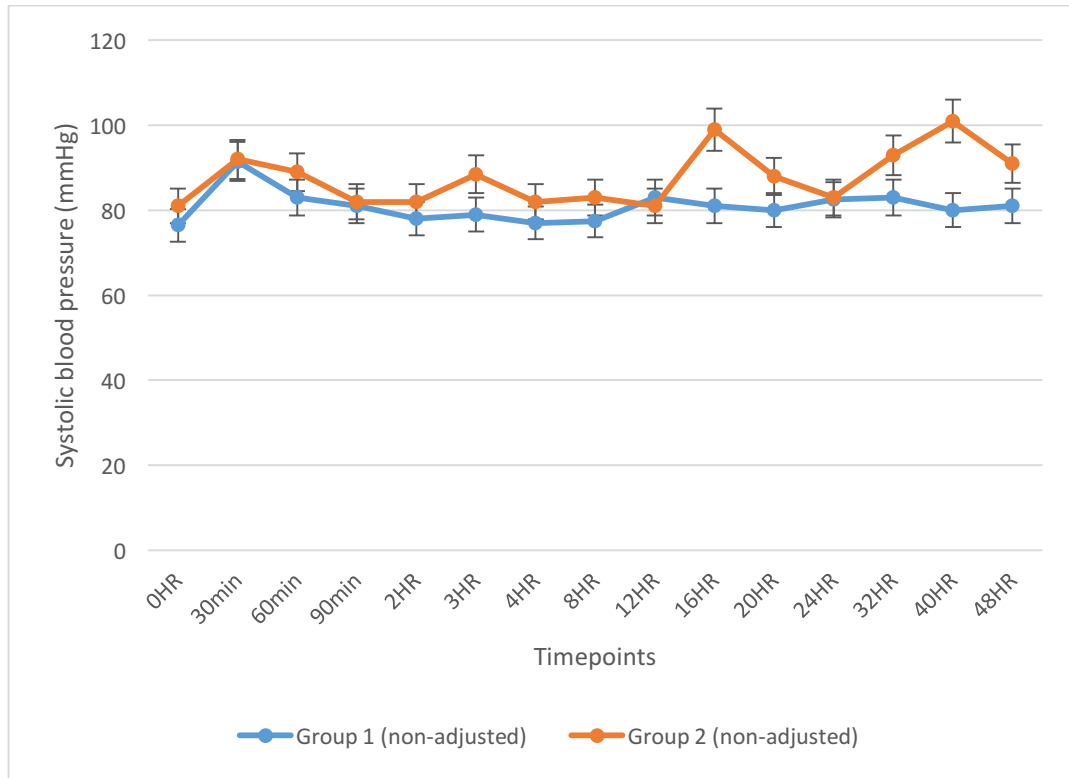

1(d)

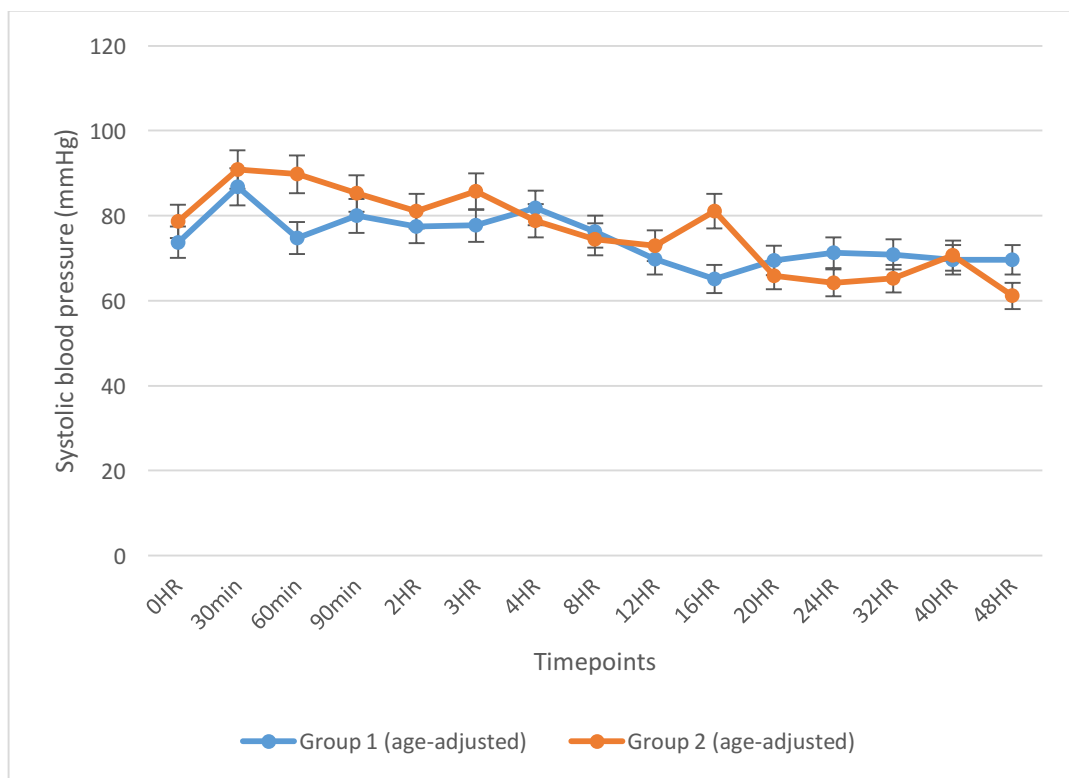

1(e)

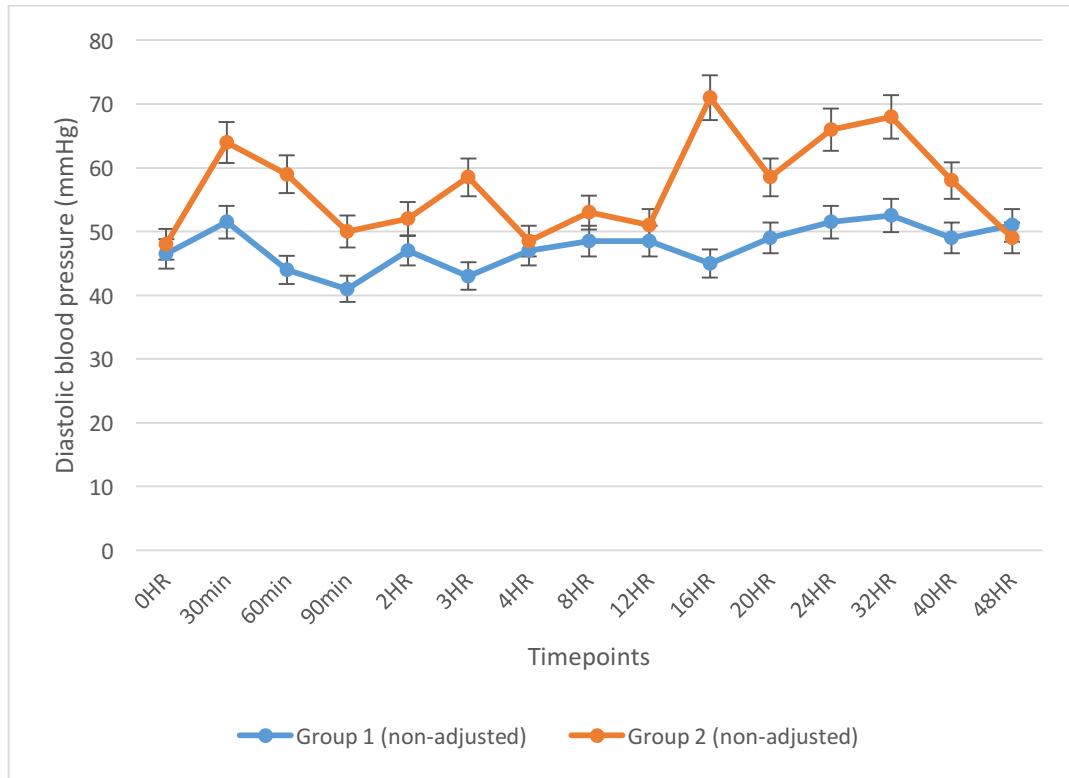

1(f)

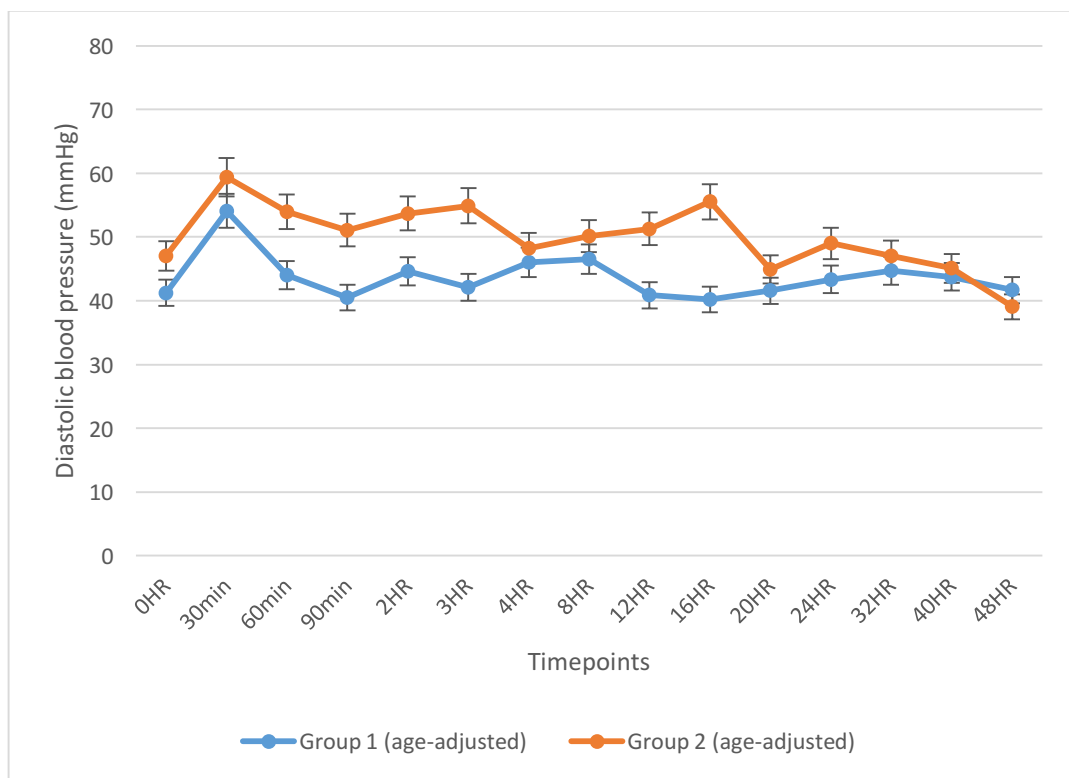

Supplement: Supplementary file 3 — By study group over time median heart rate (a) non-adjusted and (b) age-adjusted; median systolic blood pressure (c) nonadjusted and (d) age-adjusted; median diastolic blood pressure (e) nonadjusted and (f) age-adjusted. (PDF 449 kb) [file 13054_2017_1679_MOESM3_ESM.pdf]

Supplemental figure 4: Kaplan-Meier survival estimates at (a) 48 hours and (b) Day 28 by study group

(a)

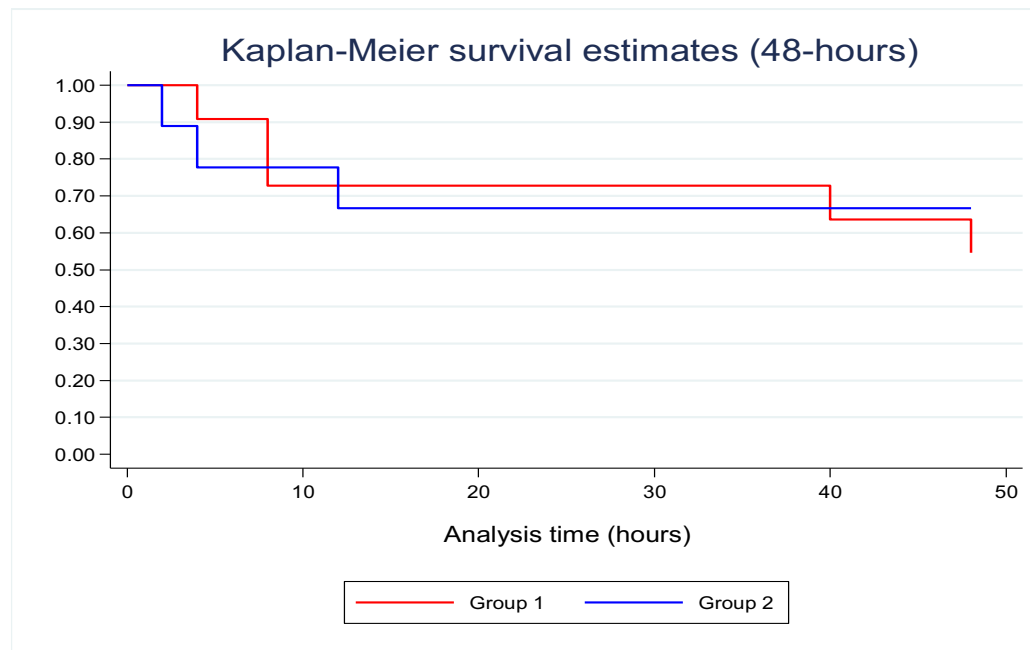

(b)

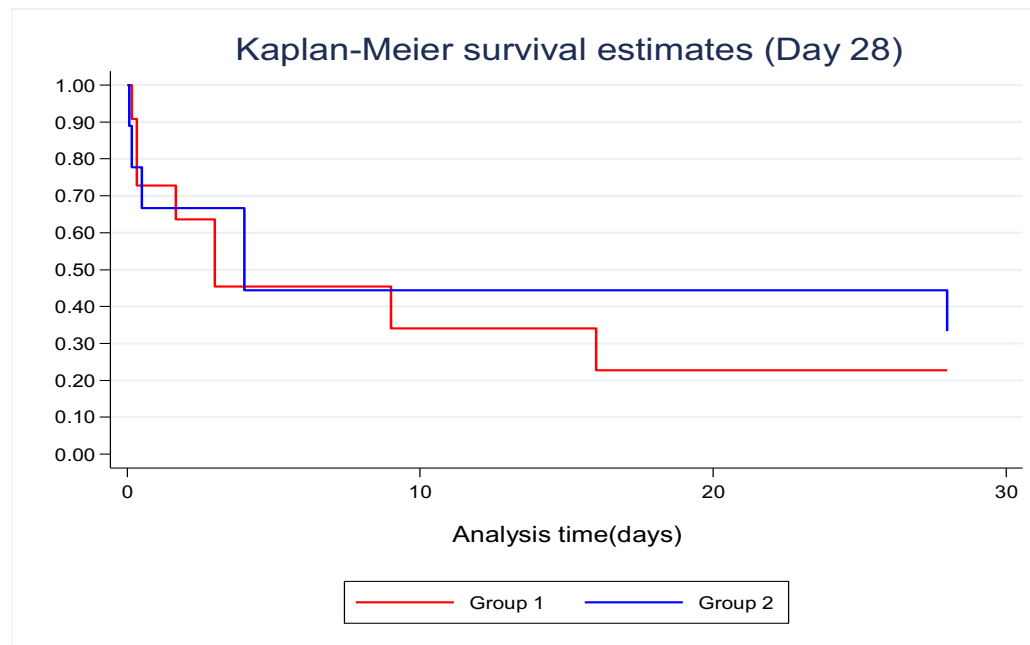

Supplement: Supplementary file 9 — Detailed narratives of the severe adverse events (SAEs). (PDF 25 kb) [file 13054_2017_1679_MOESM9_ESM.pdf]
