# Supplementary material for: Myocardial and haemodynamic responses to two fluid regimens in African children with severe malnutrition and hypovolaemic shock (AFRIM study)
Source: Crit Care. 2017 May 3;21:103. doi: 10.1186/s13054-017-1679-0 (PMC5415747; doi:10.1186/s13054-017-1679-0)
Supplement: Supplementary file 4 — Blood measurements (median and inter-quartile range) at different time points by study group. (PDF 37 kb) [file 13054_2017_1679_MOESM4_ESM.pdf]

Supplemental table 2: Blood measurements, median and inter-quartile range at different time points by study group

Parameter	Bolus + rehydration group				Rehydration-only group			
	0-hr	8-hrs	24-hrs	48-hrs	0-hr	8-hrs	24-hrs	48-hrs
**Haemoglobin (g/dl) Median, (IQR), n**	7.1 (7, 8.9), 9	6.7 (5.7, 8) 7	6.4 (5.7, 8.2), 7	7.6 (6.7, 7.7), 4	8.5 (6.4, 9.2), 9	7.4 (5.4, 8.4), 5	8.0 (7.4, 8.3), 4	8.0 (7.8,8.1), 2
Severe anaemia n, (%)	2, (22%)	2, (29%)	2, (29%)	0, (0%)	2, (22%)	3, (60%)	0, (0%)	0, (0%)
**WBC count (10^3/µL) Median, (IQR), n**	14.4 (10.6, 24.1), 9	11.3 (9.8,17.3), 9	9.5 (7.4, 12.7), 6	19.3 (15.1, 23.5), 2	10.0 (7.9, 18.9), 8	15.4 (7.4, 22.2), 8	14.1 (7.9, 24.5), 4	n/a
Leucocytosis n, (%)	6, (67%)	6, (67%)	2, (33%)	1, (50%)	2, (25%)	3, (38%)	3, (75%)	n/a
**Platelets (10^3/µL) Median, (IQR), n**	98 (73, 381), 9	116 (73,268), 7	87 (85, 290), 6	267 (261, 274), 2	87 (52, 135), 8	139 (58, 179), 6	79 (40, 119), 4	n/a
Thrombocytopenia n, (%)	5, (56%)	4, (57%)	4, (67%)	1, (50%)	6, (75%)	3, (50%)	4, (100%)	n/a
**Sodium (mmol/l) Median, (IQR), n**	127 (121, 133), 11	129 (127, 136), 9	127 (126,132), 8	127 (123, 130), 8	123 (121, 127), 8	125 (124, 125), 6	127 (124, 131), 4	130 (128, 132), 4
Hypernatraemia n, (%)	0, (0%)	0, (0%)	1, (13%)	0, (0%)	0, (0%)	0, (0%)	0, (0%)	0, (0%)
Hyponatraemia n, (%)	4, (36%)	2, (22%)	1, (13%)	1, (13%)	5, (63%)	2, (33%)	3, (75%)	0, (0%)
**Potassium (mmol/l) Median, (IQR), n**	2.4 (2.1, 3.3), 10	2.8 (2.1, 3.1), 9	2.6 (2.2, 3.1), 8	2.6 (2.2, 2.9), 8	2.8 (2.2, 5.0), 8	2.6 (2.2, 4.1), 6	3.4 (2.4, 4.5), 4	4.7 (3.9, 5.1), 4
Hypokalaemia n, (%)	4, (40%)	3, (33%)	3 (38%)	1, (13%)	3, (38%)	1, (17%)	2, (50%)	0, (0%)
**Creatinine (µmol/l) Median, (IQR), n**	63 (42, 104), 10	56 (44, 71), 8	58 (38, 87), 6	47 (35, 114), 7	65 (39, 107), 8	39 (27,93), 6	42 (34, 50), 4	44 (24, 89), 4
Hypercreatininaemia n, (%)	4, (40%)	2, (25%)	2 (33%)	2 (29%)	3, (38%)	1, (17%)	1, (25%)	0, (0%)
**Blood glucose (mmol/l) Median, (IQR), n**	6.0 (4.9, 6.8), 8	8.3 (6.3, 10.7), 4	8.6 (8.3, 9.3), 5	6.0 (4.9, 6.5), 4	2.2 (1.8, 3.9), 8	5.7 (4.2, 8.7), 5	7.2 (7, 7.3), 2	4.7 (4.1, 5.3), 2
Hypoglycaemia n, (%)	1, (13%)	1, (25%)	0, (0%)	0, (0%)	5, (63%)	2, (40%)	0, (0%)	0, (0%)
**Lactate (mmol/l) Median, (IQR), n**	2.9 (1.4, 5.2), 9	1.7 (1.3, 5.9), 9	2.1 (1.5, 3.7), 8	2.7 (1.9, 2.9), 5	2.2 (1.9, 3.6), 9	2.0 (1.7, 2.5) 6	3.1 (2.4, 4.2), 3	1.2 (1.1, 1.4), 4
Hyperlacataemia n, (%)	4, (44%)	3, (33%)	3, (38%)	1, (20%)	3, (33%)	2, (33%)	2, (67%)	0, (0%)

Severe anaemia (haemoglobin <5g/dL); leucocytosis (white blood cell count >12.5 x10^3/µL); thrombocytopenia (platelet count < 159 x10^3/µL); severe hyponatraemia (sodium level <125mmol/L); severe hypokalaemia (potassium level <2.5mmol/L); hypercreatinaemia (creatinine level $\geq$ 80µmol/L); hypoglycaemia (blood glucose level <3mmol/L); hyperlacataemia (lactate level $\geq$ 3mmol/L).
