# Supplementary material for: Myocardial and haemodynamic responses to two fluid regimens in African children with severe malnutrition and hypovolaemic shock (AFRIM study)
Source: Crit Care. 2017 May 3;21:103. doi: 10.1186/s13054-017-1679-0 (PMC5415747; doi:10.1186/s13054-017-1679-0)
Supplement: Supplementary file 5 — Blood test parameters. a Lactate. b Haemoglobin. c Potassium. d Sodium. e White blood cell count. f Creatinine. (PDF 59 kb) [file 13054_2017_1679_MOESM5_ESM.pdf]

Supplemental Figure 2: Blood test parameters (a) Lactate, (b) Haemoglobin, (c) Potassium, (d) Sodium, (e) White blood cell count, (f) Creatinine

(a)

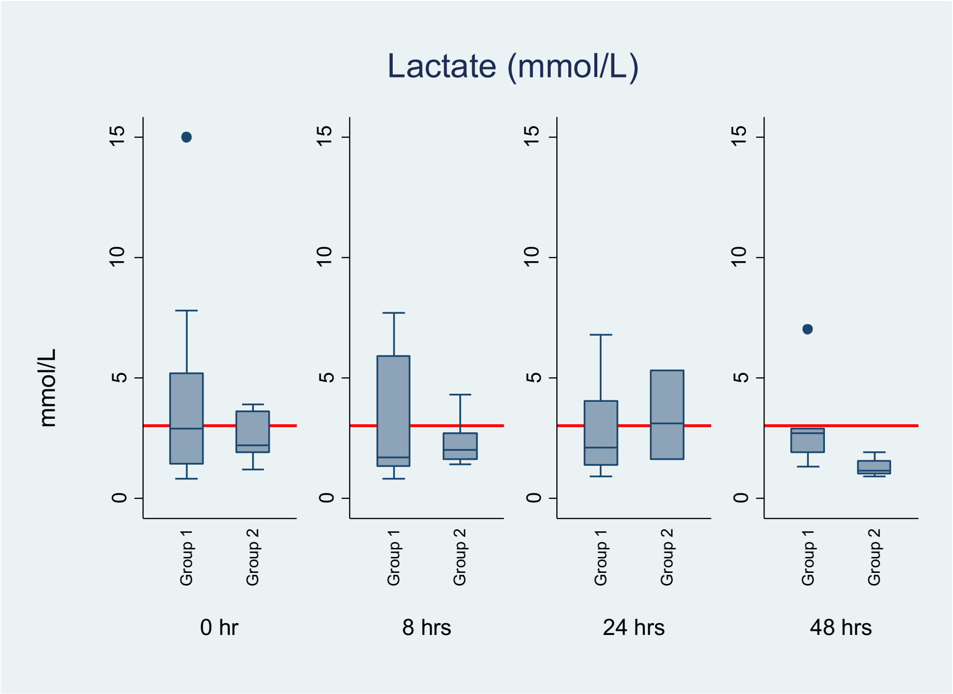

(b)

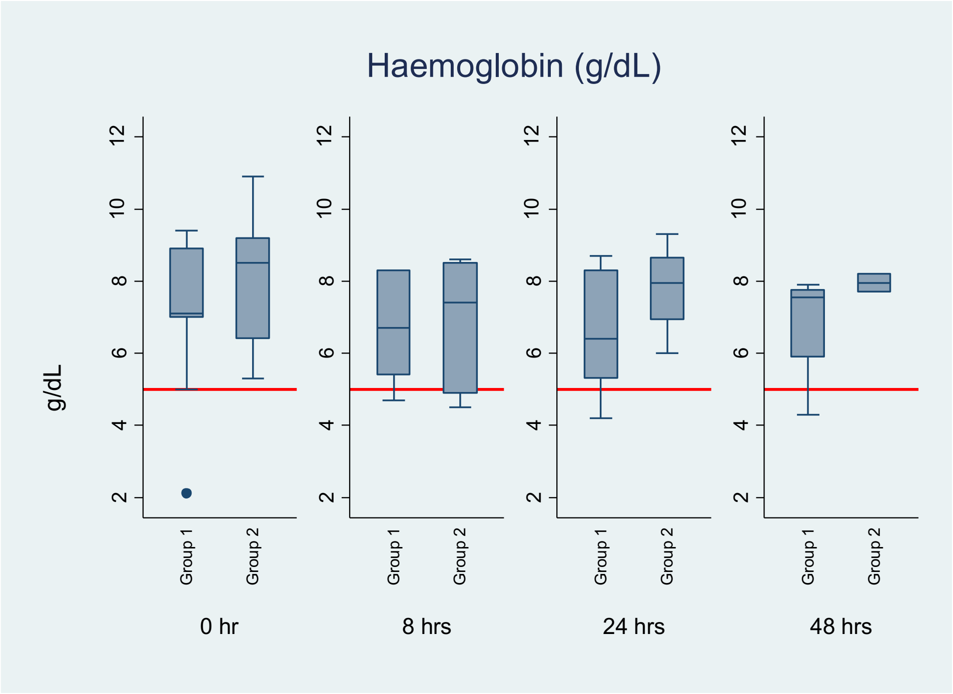

Blood test parameters	0-hour		8-hours		24-hours		48-hours	
	Group 1	Group 2	Group 1	Group 2	Group 1	Group 2	Group 1	Group 2
n	11	9	11	7	8	5	6	5

(c)

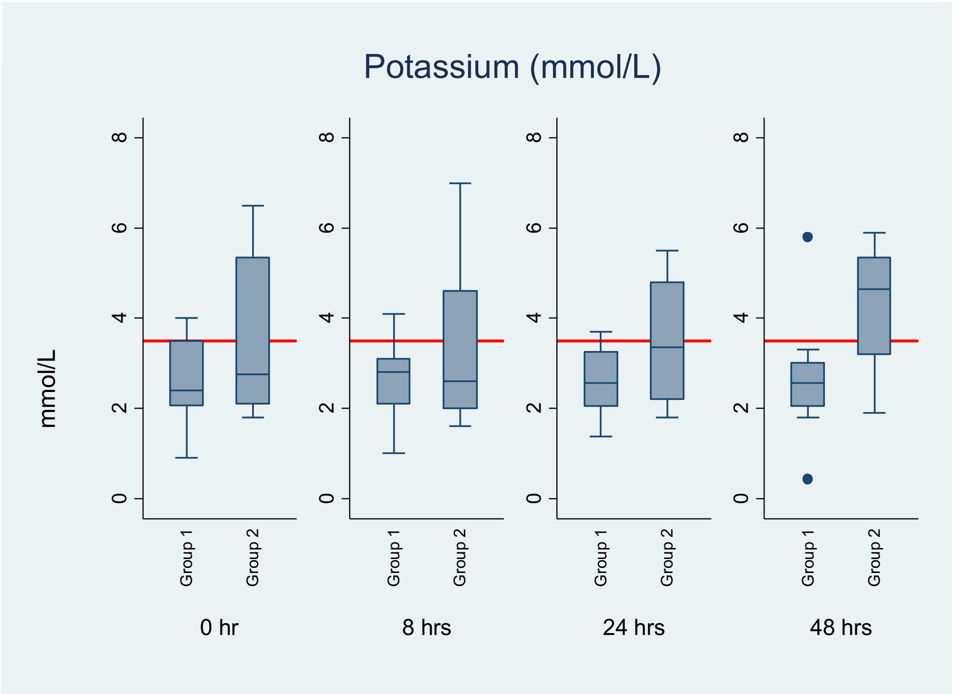

(d)

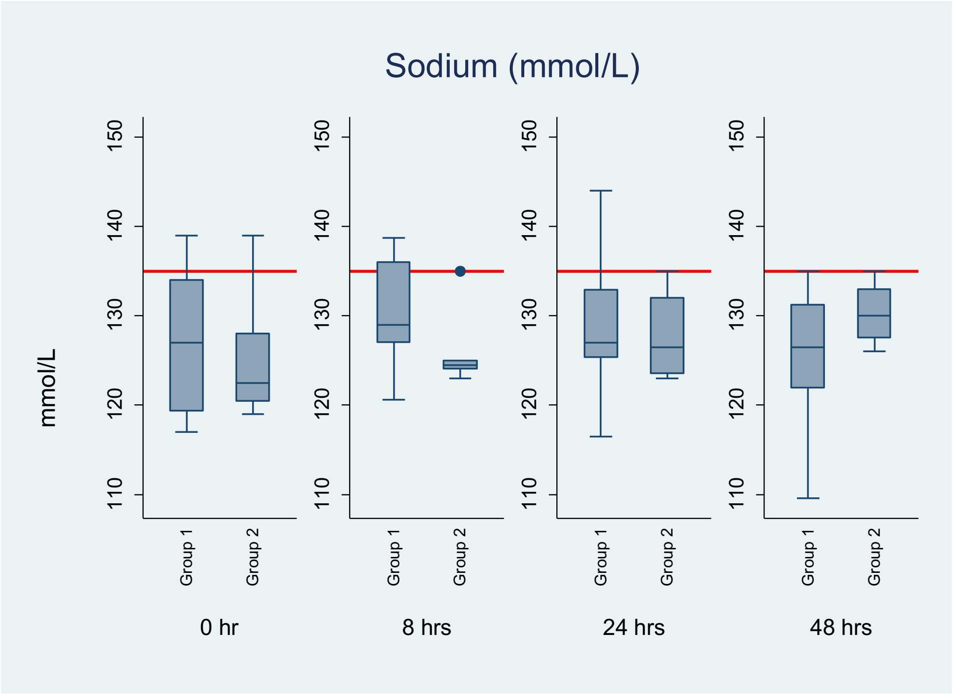

Blood test parameters	0-hour		8-hours		24-hours		48-hours	
	Group 1	Group 2	Group 1	Group 2	Group 1	Group 2	Group 1	Group 2
n	11	9	11	7	8	5	6	5

(e)

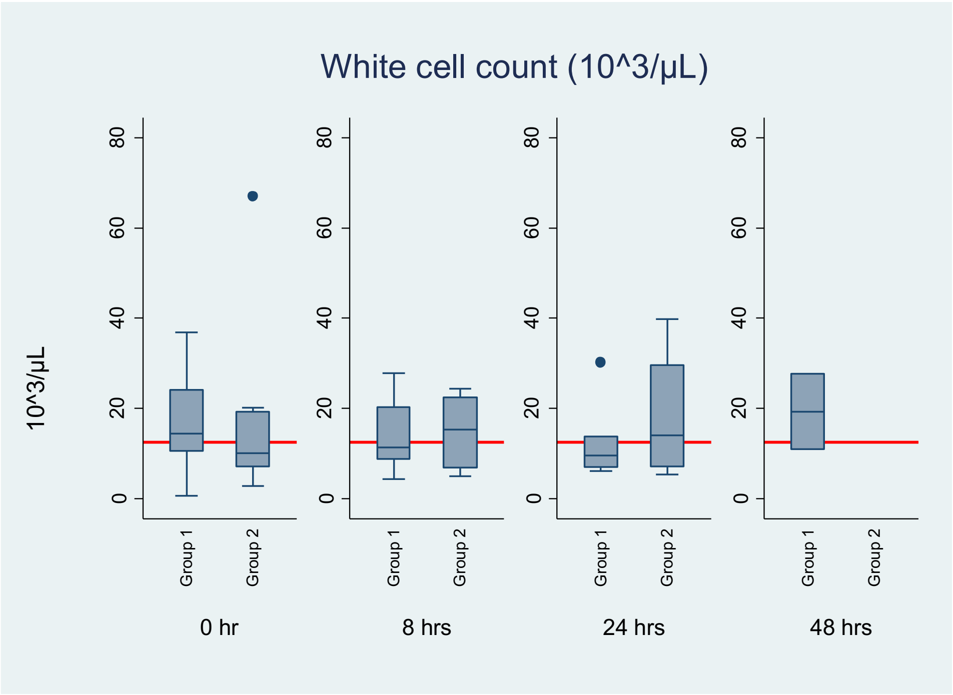

(f)

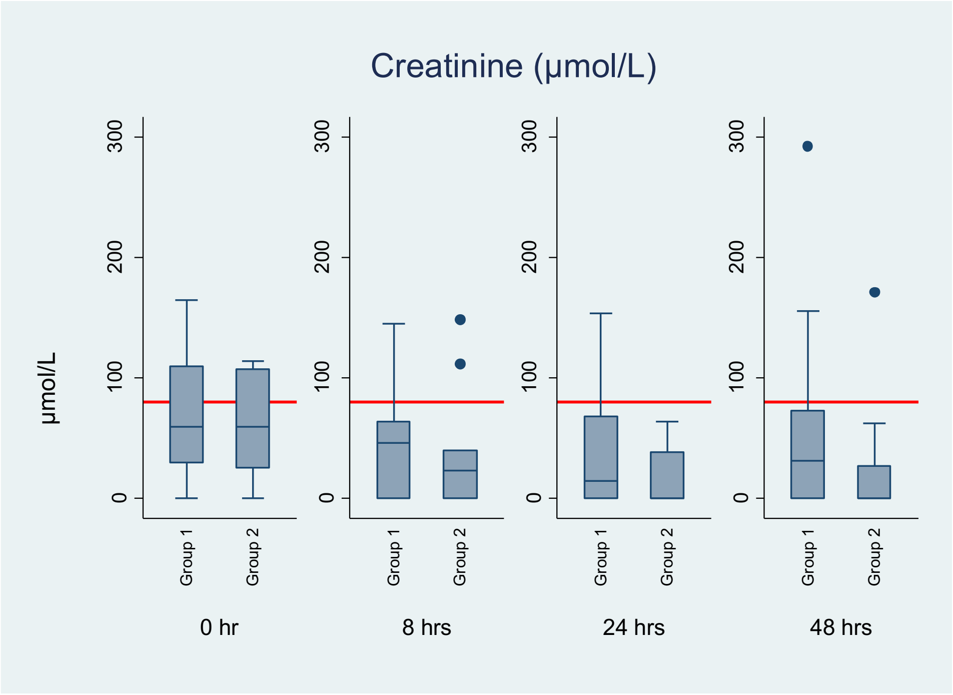

Blood test parameters	0-hour		8-hours		24-hours		48-hours	
	Group 1	Group 2	Group 1	Group 2	Group 1	Group 2	Group 1	Group 2
n	11	9	11	7	8	5	6	5
