# Supplementary material for: Myocardial and haemodynamic responses to two fluid regimens in African children with severe malnutrition and hypovolaemic shock (AFRIM study)
Source: Crit Care. 2017 May 3;21:103. doi: 10.1186/s13054-017-1679-0 (PMC5415747; doi:10.1186/s13054-017-1679-0)
Supplement: Supplementary file 6 — Cardiac haemodynamic parameters. a Fractional shortening. b Cardiac index. c Left ventricle end-diastolic volume index. d Systemic vascular resistance index. e Inferior vena cava collapsibility index. (PDF 19 kb) [file 13054_2017_1679_MOESM6_ESM.pdf]

Supplemental table 3: Cardiac bio-markers medians, inter-quartile ranges and standard deviation at admission and 48-hours by study group

**Parameter**	**Bolus + rehydration group**						**Rehydration-only group**					
	**Pre-fluid**			**48-hour**			**Pre-fluid**			**48-hour**		
	N	Median, (IQR)	Std.dev	n	Median, (IQR)	Std.dev	n	Median, (IQR)	Std.dev	n	Median, (IQR)	Std.dev
BNP* (pg/ml)	10	336 (131, 540)	520	5	322 (317, 359)	437	9	168 (117, 214)	76	3	0 (0, 162)	93
BNP > 300pg/ml n, (%)	6	(60%)		4	(57%)		2	(22%)		0	(0%)	
Troponin I* (pg/ml)	10	0 (0, 443)	327	5	0 (0, 369)	165	3	0 (0, 370)	336	3	0 (0, 399)	230

BNP, brain natriuretic peptide; IQR, inter-quartile range

*BNP and Troponin I measurements were sampled at admission (baseline) and 48 hours only
