# Supplementary material for: Myocardial and haemodynamic responses to two fluid regimens in African children with severe malnutrition and hypovolaemic shock (AFRIM study)
Source: Crit Care. 2017 May 3;21:103. doi: 10.1186/s13054-017-1679-0 (PMC5415747; doi:10.1186/s13054-017-1679-0)
Supplement: Supplementary file 7 — Cardiac bio-markers (medians, interquartile ranges and standard deviation) at admission and 48 h, by study group. (DOC 74 kb) [file 13054_2017_1679_MOESM7_ESM.doc]

Supplemental Figure 3: Cardiac haemodynamic parameters (a) Fractional shortening, (b) Cardiac index, (c) Left ventricle end-diastolic volume index, (d) Systemic vascular resistance index, (e) Inferior vena caval collapsibility index

(a)

| Fractional shortening, FS (%) | Pre-fluid | | Post-fluid | | 24-hours | |
| --- | --- | --- | --- | --- | --- | --- |
|  | Group 1 | Group 2 | Group 1 | Group 2 | Group 1 | Group 2 |
| N | 11 | 9 | 11 | 7 | 8 | 5 |
| Median (IQR) | 28  (23,31) | 31  (23,33) | 30  (24,32) | 31  (27,35) | 29  (28,31) | 28  (25,29) |

(b)

| Cardiac index, CI (L/min/m^2^) | Pre-fluid | | Post-fluid | | 24-hours | |
| --- | --- | --- | --- | --- | --- | --- |
|  | Group 1 | Group 2 | Group 1 | Group 2 | Group 1 | Group 2 |
| N | 11 | 9 | 11 | 7 | 7 | 5 |
| Median  (IQR) | 7  (4,8) | 7  (5,9) | 6  (5,8) | 7  (5,8) | 5  (5,6) | 6  (6,7) |

(c)

| LV-EDVI (ml/m^2^) | Pre-fluid | | Post-fluid | | 24-hours | |
| --- | --- | --- | --- | --- | --- | --- |
|  | Group 1 | Group 2 | Group 1 | Group 2 | Group 1 | Group 2 |
| n | 11 | 9 | 11 | 7 | 7 | 5 |
| Median  (IQR) | 60  (48,77) | 50  (48,59) | 62  (52,76) | 58  (51,60) | 58  (44,64) | 74  (58,76) |

(d)

| SVRI (ds/cm^5^/m^2^) | Pre-fluid | | Post-fluid | | 24-hours | |
| --- | --- | --- | --- | --- | --- | --- |
|  | Group 1 | Group 2 | Group 1 | Group 2 | Group 1 | Group 2 |
| N | 11 | 9 | 11 | 7 | 7 | 5 |
| Median  (IQR) | 1349  (1039, 1797) | 1265  (1155, 1969) | 1275  (1189, 1610) | 1105  (1071, 1655) | 1422  (1294, 1556) | 2102  (1883, 2113) |

(e)

| IVC CI (%) | Pre-fluid | | Post-fluid | | 24-hours | |
| --- | --- | --- | --- | --- | --- | --- |
|  | Group 1 | Group 2 | Group 1 | Group 2 | Group 1 | Group 2 |
| n | 10 | 8 | 10 | 7 | 8 | 5 |
| Median  (IQR) | 30  (23,46) | 38  (28,43) | 38  (37,39) | 28  (24,35) | 34  (23,39) | 23  (22,26) |
