# Supplementary material for: Myocardial and haemodynamic responses to two fluid regimens in African children with severe malnutrition and hypovolaemic shock (AFRIM study)
Source: Crit Care. 2017 May 3;21:103. doi: 10.1186/s13054-017-1679-0 (PMC5415747; doi:10.1186/s13054-017-1679-0)
Supplement: Supplementary file 8 — Kaplan-Meier survival estimates at 48 h (a) and day 28 (b), by study group. (DOCX 35 kb) [file 13054_2017_1679_MOESM8_ESM.docx]

Supplemental table 4: Severe adverse events (SAEs) review

| **Adjudication** Whether there was a causative relationship between the reported event and the fluid resuscitation given  **Question 1** Whether fatal and non-fatal events could be related to fluid resuscitation?  **Question 2** Was there a causative relationship between the reported event and the fluid resuscitation?  **Additional comments** on main cause of death and possible diagnosis   \| **SAE review** \| **Description** \| \| --- \| --- \| \| **Unrelated** \| There is no evidence of any causal relationship \| \| **Unlikely** \| There is little evidence to suggest there is a causal relationship (e.g. the event did not occur within a reasonable time after administration of the trial medication). There is another reasonable explanation for the event (e.g. the participant’s clinical condition, other concomitant treatment). \| \| **Possible** \| There is some evidence to suggest a causal relationship (e.g. because the event occurs within a reasonable time after administration of the trial medication). However, the influence of other factors may have contributed to the event (e.g. the participant’s clinical condition, other concomitant treatments). \| \| **Probable** \| There is evidence to suggest a causal relationship and the influence of other factors is unlikely. \| \| **Definitely** \| There is clear evidence to suggest a causal relationship and other possible contributing factors can be ruled out. \| \| **Not assessable** \| There is insufficient or incomplete evidence to make a clinical judgement of the causal relationship. \| | | | |
| --- | --- | --- | --- | --- | --- | --- | --- | --- | --- | --- | --- | --- | --- | --- | --- | --- | --- |
|  | **SAE review** | **Clinical details** | **Group** |
| **AFR 301**  Fatal | **Unrelated**  1/ Not related to bolus fluid and volume administered  Fatality related to severity of illness (i.e. severe malnutrition and untreated HIV co-morbidity) and possible terminal aspiration pneumonitis | **Clinical History and admission findings**  A severely malnourished child weighing 6.1kg; WHZ-score -4.9; mid-upper arm circumference (MUAC) 9.5cm, with generalized oedema (kwashiorkor) presented with a 4-day history of gastroenteritis, fever and cough.  Admitted with severe dehydration and severe WHO shock as the child had failed oral rehydration.  On physical examination there were all four features of shock: prolonged capillary refill time (CRT)> 3 seconds, cold peripheries, temperature gradient, a weak and rapid radial pulse and persistent prostration (impaired consciousness despite hypoglycaemia correction).  Admission blood tests: severe hypoglycaemia (blood sugar 0.9mmol/L), hyponatraemia (sodium 127 mmol/L), hypokalaemia (potassium 0.9mmol/L), haemoglobin 7.1d/dL, leukocytopenia (white cell count 3,400/μL), thrombocytopenia (platelets 98,000/μL), a negative malaria rapid diagnostic test (RDT), HIV positive but the patient was not on anti-retroviral treatment. BNP 23.3pg/ml and troponin I level was 0.  Initial treatment: Blood sugar correction (5ml/kg of 10% dextrose) and intravenous antibiotics (1^st^ line ampicillin and gentamicin). Fluid resuscitation with two boluses of Ringer’s lactate (RL) 15ml/kg body weight each over 1 hour.  Echocardiographic findings: Fractional shortening (FS) before bolus fluid was 27% that reduced to 23% after bolus fluid but rose to 34% by 24 hours. Inferior vena caval collapsibility index (IVCCI) gradually reduced from 43% before bolus fluid to 37% after bolus fluid and 18% by 24 hours as expected. The systemic vascular resistance index (SVRI) was high 2,431 dscm^-5^/m^2^ before bolus fluid and 1,718 dscm^-5^/m^2^ after bolus fluid while the stroke volume index (SVI) reduced from 44ml/m^2^ before bolus fluid to 36ml/m^2^ after bolus fluid.  Clinical progress:  Initial improvement after two fluid boluses (RL, 15ml/kg/hr); the patient then received maintenance fluid at 4ml/kg/hr. Had good urine output (4.6 ml/kg/hr). Switched to oral intake after 24 hours and tolerated well. Repeat blood electrolyte testing showed minimal improvement at 8 hours (sodium 129mmol/L and potassium 1.0mmol/L) and at 24 hours (sodium 127mmol/L and potassium 1.8mmol/L). Patient was sick but stabilized from shock and started on standard malnutrition treatment oral feeds at 24 hours. There was a sudden deterioration in clinical condition at 48 hours while the patient was taking oral F75 therapeutic milk formulation due to possible aspiration. Developed sudden cough, oral feeds were stopped and restarted on intravenous maintenance fluids at 4ml/kg/hr. Blood electrolytes were rechecked (sodium 129mmol/L and potassium 2.6mmol/L). The patient remained sickly with monitoring in the high dependency unit (HDU), did not relapse into shock and was on maintenance fluid but developed asystole and resuscitation attempts were unsuccessful. Died 68 hours after admission and therefore classified as a late death.  **Attending clinician’s diagnosis**: Death resulting from cardio-respiratory arrest and possible aspiration pneumonitis. The child was very severely malnourished and sero-exposed but not on anti-retroviral treatment.  **End-point review:** Relationship between fluid boluses and cause of mortality is unlikely. | **Group 1**  **(bolus + maintenance)** |
| **AFR 401**  Fatal | **Possible**  Received bolus fluid volume 20ml/kg/hr instead of 15ml/kg/hr but had no signs of fluid overload  Fatality related to severity of illness: acidosis, hypoxia and multiple organ failure | **Clinical History and admission findings**  A severely malnourished child weighing 8kg (WHZ-score > -3) with kwashiorkor and bipedal oedema presented with a short history of diarrhoea, vomiting, fever and respiratory distress.  Physical examination at admission showed the following features of shock: cold peripheries, temperature gradient, weak and rapid radial pulse and lethargy. Patient was hypothermic (temperature 34.8^0^C), severely hypoxic (oxygen saturation 65% in room air), tachypnoeic (respiratory rate 39 breaths/min), tachycardic (heart rate 129 beats/min), severely decreased skin turgor with a prolonged return of skin pinch (5 seconds) and a capillary refill time (CRT) of 2 seconds. Chest auscultation revealed bilateral coarse crackles (severe pneumonia), no evidence of heart failure (no gallop rhythm or distension of neck veins). Patient had hepatomegaly 5 cm below costal margin (BCM) and cheilitis.  Admission blood tests: normal sodium 139mmol/L and potassium 3.9mmol/L. High lactate 5.2mmol/L and unrecordable blood sugar. Both malaria rapid diagnostic and HIV tests were negative. BNP 101.5pg/ml and troponin I 591.3pg/ml.  Initial treatment: Oxygen supplementation, kept warm, glucose (5ml/kg of 10% dextrose) and intravenous antibiotics (2^nd^ line chloramphenicol). Fluid resuscitation with two boluses of RL 20ml/kg body weight each over 1 hour, (instead of 15ml/kg body weight). However, at no point was there evidence of fluid overload.  Echocardiographic findings: Fractional shortening (FS) before bolus fluid was 18%, but increased to 30% after bolus fluid. The systemic vascular resistance index (SVRI) was high 1,103 dscm^-5^/m^2^ before bolus fluid and 1,214 dscm^-5^/m^2^ after bolus fluid while the stroke volume index (SVI) increased from 30ml/m^2^ before bolus fluid to 34ml/m^2^ after bolus fluid. The inferior vena cava was barely visible before and after fluid administration.  Clinical progress:  The patient had an initial slight improvement after two fluid boluses (RL, 20ml/kg/hr) and received maintenance fluid at 4ml/kg/hr. The patient remained acidotic (lactate 6.7mmol/L), hypoxic (severe pneumonia), oliguric (creatinine rose from 88 μmol/L at admission to 94μmol/L pre-terminal) i.e. passed 4.5ml/kg/hr of loose stool (by weighed diapers) and died 8 hours after admission.  **Attending clinician’s diagnosis**: Death to be due to multiple organ failure as a consequence of lactic acidosis and hypoxia caused by the severity of the disease process and severe malnutrition.  **End-point review**: Severe shock, acidosis and metabolic derangement, severe pneumonia and dehydrating diarrhoea with large volume stool outcome. Echo finding suggested slight improvement in the FS and SVI after fluid administration but the SVRI remained persistently high. Oliguria may have indicated established acute kidney injury. Classified as early death occurring 8 hours after admission. | **Group 1**  **(bolus + maintenance)** |
| **AFR 402**  Fatal | **Unrelated**  1/ Not related to bolus fluid and volume administered  Fatality related to severity of illness: severe malnutrition and multiple organ failure | **Clinical History and admission findings**  A severely malnourished child weighing 5.5kg (WHZ > -3; MUAC 12.4cm), with generalized oedema (kwashiorkor) with a history of diarrhoea, fever and respiratory distress.  Admitted in shock (i.e. cold peripheries with a temperature gradient, weak and rapid radial pulse and lethargy).  Physical examination at admission: The patient was prostrate, heart rate 98 beats/min, respiratory rate 27 breaths/min, deep breathing but not hypoxic (oxygen saturation 98% in room air), had skin desquamation on the scalp. On auscultation, the chest was clear with no crackles and no evidence of heart failure (no gallop rhythm or distension of neck veins).  Admission blood tests: Severe hyponatraemia (sodium 126mmol/L), potassium 4.0mmol/L, creatinine 59µmol/L, lactate 1.4 mmol/L. Both malaria rapid diagnostic and HIV tests were negative. BNP 345.6pg/ml and troponin I level was 0.  Initial treatment: The patient received two fluid boluses of RL 15ml/kg body weight each over 1 hour.  Intravenous antibiotics 2^nd^ line ceftriaxone with added cloxacillin (due to the skin desquamation).  Echocardiographic findings: Fractional shortening (FS) before bolus fluid was 21%, increased slightly to 25% after bolus fluid but rose to 39% by 24 hours. Inferior vena caval collapsibility index (IVCCI) unexpectedly increased from 14% before bolus fluid to 39% immediately after bolus fluid and reduced to 13% by 24 hours. The systemic vascular resistance index (SVRI) was high 1,349 dscm^-5^/m^2^ before bolus fluid and 1,616 dscm^-5^/m^2^ after bolus fluid while the stroke volume index (SVI) remained unchanged at 25% after fluid bolus.  Clinical progress:  The patient improved after the initial two boluses (RL, 15ml/kg/hr) and thereafter received maintenance fluid at 4ml/kg/hr for a total of 7 hours then switched to oral intake as tolerated. The patient had been oliguric for the first 8 hours passing only 1.5ml/kg/hr of loose watery stools (by weighed diapers) but thereafter urine output improved to normal 3ml/kg/hr. Over the course of admission, the patient received two blood transfusions (10ml/kg each) for anaemia (clinically pale).  **Attending clinician’s diagnosis**: The patient died after 15 days while on standard treatment for severe malnutrition. No further fluid boluses were given during the hospitalization. Death due to multiple organ failure as a consequence of the disease process and severe malnutrition.  **End-point review**: The patient recovered from the initial shock following resuscitation with bolus (RL, 15ml/kg/hr) and received nutritional rehabilitation with F75 therapeutic milk formulation but showed minimal improvement in the skin desquamation over the course of admission. Mortality due to severe malnutrition and unlikely to be related to fluids. Classified as a late death. | **Group 1**  **(bolus + maintenance)** |
| **AFR 403**  Fatal | **Unrelated**  1/ Not related to bolus fluid and volume administered  Fatality related to severe respiratory distress and multiple organ failure | **Clinical History and admission findings**  A severely malnourished child weighing 7.6kg (WHZ-score > -3; MUAC 11.6cm) was admitted in shock (i.e. comatose, cold peripheries with a temperature gradient, weak and rapid radial pulse, CRT 2 seconds) with a history of fever, diarrhoea, vomiting, cough and respiratory distress.  Clinical examination at admission, the patient was comatose, had bipedal oedema with skin desquamation over the upper and lower limbs, heart rate 100 beats/min, respiratory rate 37 breaths/min, deep breathing and chest wall in-drawing (oxygen saturation 92 % in room air). There was hepatomegaly of 6.5cm below the right costal margin but no signs of heart failure (i.e. chest was clear on auscultation with no crackles, no gallop rhythm and no distension of neck veins).  Admission blood tests: Very severe hyponatraemia (sodium 117mmol/L), hypokalaemia (potassium level was unrecordably low) and leucocytosis (white cell count 16,300/μL). Lactate was mildly elevated (3.0mmol/L) but the rest of the tests were within normal limits i.e. blood sugar 5.2 mmol/L, haemoglobin 9.8 g/dL, creatinine 21μmol/L. Malaria rapid diagnostic test and HIV test were both negative. BNP 327.8pg/ml and troponin I 443.2pg/ml.  Initial treatment: Patient received two boluses (RL, 15ml/kg/hr) and ceftriaxone intravenous antibiotics.  Echocardiographic findings: Fractional shortening (FS) before bolus fluid was 28%, increased to 34% immediately after bolus fluid but settled back at 29% by 24 hours. Inferior vena caval collapsibility index (IVCCI) unexpectedly increased from 17% before bolus fluid to 36% and remained high (36%) at 24 hours. The systemic vascular resistance index (SVRI) was persistently high 1,797 dscm^-5^/m^2^ before bolus fluid and 1,603 dscm^-5^/m^2^ immediately after bolus fluid and 1,863 dscm^-5^/m^2^ at 24 hours, while the stroke volume index (SVI) increased marginally from 21% to 24 % after fluid bolus.  Clinical progress:  The patient showed initial improvement after the 2 boluses and thereafter received maintenance fluid at 4ml/kg/hr for a total of 20 hours; had good urine output of 3ml/kg/hr in the first 24 hours and was then switched to oral rehydration (RESOMAL) and potassium supplementation (potassium levels rose from unrecordable at admission to 1.74mmol/L at 8 hours and was 2.72mmol/L by 48 hours). The patient received standard treatment for severe malnutrition i.e. F75 therapeutic milk formulation. The patient remained sickly over the course of admission but no additional fluid boluses were administered as the patient did not relapse into shock. The patient remained septic on sixth day of admission despite treatment (persistently high white cell count, 13,700/μL and lactate levels, 7mmol/L). The guardian attempted to feed cow’s milk per oral on the seventh day and the patient aspirated, developed respiratory distress and went into an asystole arrest. Resuscitation efforts were unsuccessful.  **Attending clinician’s diagnosis**: Patient died after 7 days. Death to be due to severe respiratory distress, from aspiration pneumonitis and multiple organ failure.  **Endpoint review group** suggested mortality was unrelated to boluses and due to uncorrected sepsis, possible aspiration pneumonia and severe oedematous malnutrition but there was no evidence of heart failure. Classified as a late death (7 days into admission). | **Group 1**  **(bolus + maintenance)** |
| **AFR 405**  Fatal | **Unrelated**  1/ Not related to bolus fluid and volume administered  Fatality related to respiratory arrest in severe malnutrition with metabolic derangements and likely aspiration | **Clinical History and admission findings**  A severely malnourished child weighing 10kg (WHZ-score > -3) with generalized oedema (kwashiorkor) was admitted with severe dehydration and shock (i.e. cold peripheries with a temperature gradient, weak and rapid radial pulse and a prolonged capillary refill time (CRT) 4 seconds with a history of diarrhoea, vomiting and difficulty breathing.  Clinical examination at admission: The patient had prostration, deep breathing and in-drawing but not hypoxic (oxygen saturation 99 % in room air), respiratory rate 18 breaths/min and relative bradycardia (heart rate 107 beats/min). Prolonged return of skin pinch (4 seconds) and hepatomegaly of 4cm below the right costal margin but no signs of heart failure (i.e. clear chest with no crackles on auscultation, no gallop rhythm and neck veins were not distended.  Admission blood tests: severe hyponatraemia (sodium 122mmol/L), hypokalaemia (2mmol/L), creatinine 57 μmol/L. Leukocytosis (white cell count 14,400/μL), haemoglobin 7.6g/dL, Malaria rapid diagnostic test was positive. HIV test was not done. BNP 131.1pg/ml and troponin I level was 0.  Initial treatment: Fluid resuscitation with two fluid boluses (RL, 15ml/kg/hr). Anti-malarial treatment and intravenous antibiotics (ampicillin and gentamicin).  Echocardiographic findings: Fractional shortening (FS) remained unchanged after (34%), and dropped marginally to 32 by 24 hours. Inferior vena caval collapsibility index (IVCCI) unexpectedly increased from 29% to 33% after bolus fluid administration but had reduced to 20% at 24 hours. The systemic vascular resistance index (SVRI) which was within normal range for age before fluid bolus reduced after fluid bolus (i.e. 683 dscm^-5^/m^2^ and 569 dscm^-5^/m^2^ respectively), while the stroke volume index (SVI) increased from 30% to 40 % after fluid bolus.  Clinical progress:  The patient had a urine output of 1.25ml/kg/hr in the first 24 hours despite large volumes of watery diarrhoea (volume 1,475mls by diaper weight) and received maintenance fluid (HSD/D5%) at 4ml/kg/hr after the initial 2 boluses (RL, 15ml/kg/hr) and started on F75 milk formulation for severe malnutrition treatment. While in the ward, the haemoglobin level dropped from admission level of 7.6g/dL to 6g/dL and the patient received blood transfusion (10ml/kg over 3 hours) which raised the haemoglobin level to 9.5g/dL on the second day of admission. Three days into admission, the patient remained severely hyponatraemic (sodium 125mmol/L) and hypokalaemic (potassium 2.5mmol/L) and developed cardio-respiratory arrest. Resuscitation attempts were unsuccessful, the pre-terminal lactate level was elevated (5mmol/L).  **Attending clinician’s diagnosis**: Patient died after 72 hours and was found to have excessive secretions. Death likely to be due to respiratory arrest and possible aspiration pneumonitis.  **End-point review group**, the patient died from metabolic derangements, and uncorrected sepsis in severe oedematous malnutrition with possible aspiration pneumonia. There were no signs suggestive of volume overload or heart failure. Classified as a late death occurring after 72 hours. | **Group 1**  **(bolus + maintenance)** |
| **AFR 406**  Fatal | **Unrelated**  1/ Not related to bolus fluid and volume administered  Fatality related to severity of illness: acidosis, hypoxia, acute renal and multiple organ failure | **Clinical History and admission findings**  A severely malnourished child weighing 7kg (WHZ > -3) was admitted in shock (i.e. cold peripheries with a temperature gradient, weak and rapid radial pulse and CRT> 4 seconds). Had a 5-day history of diarrhoea, vomiting and cough prior to admission.  Clinical examination: The patient had prostration, bradycardia (heart rate 74 beats/min), severe respiratory distress (i.e. Kussmaul’s breathing, tachypnoea-respiratory rate 60 breaths/min and hypoxia-oxygen saturation 77 % in room air), skin and hair changes of malnutrition but non-oedematous (i.e. marasmic) and hepatomegaly of 4cm below the right costal margin but not in heart failure (i.e. clear chest with no crackles on auscultation, no gallop rhythm and no distension of neck veins).  Admission blood tests: Very severe hyponatraemia (sodium 119mmol/L), hypokalaemia (potassium 2.7mmol/L), elevated creatinine (126 μmol/L), and severe lactaemia (lactate 15 mmol/L.), Leukocytopenia (white cell count 600/μL) and thrombocytopenia (platelets 46,000/μL), blood glucose 6.3 mmol/L and haemoglobin 9.1 g/dL. Positive malaria rapid diagnostic test (RDT), HIV test was not done. BNP 1,207.8pg/ml and troponin I level was 0.  Initial treatment: Supplemental oxygen and received two boluses (RL, 15ml/kg/hr). Intravenous antibiotics (ampicillin and gentamicin) and anti-malarial treatment were initiated.  Echocardiographic findings: Fractional shortening (FS) before bolus fluid was 29%, reduced to 24% immediately after bolus fluid. Inferior vena caval collapsibility index (IVCCI) gradually reduced from 58% before bolus fluid to 49% after bolus fluid as expected. The systemic vascular resistance index (SVRI) was high 1,796 dscm^-5^/m^2^ before bolus fluid and unexpectedly rose to 2,798 dscm^-5^/m^2^ after bolus fluid while the stroke volume index (SVI) reduced from 28ml/m^2^ before bolus fluid to 15ml/m^2^ after bolus fluid.  Clinical progress:  The patient remained oliguric, passing 1.4ml/kg/hr of loose watery stools (by diaper weight) during the following hours. Maintenance fluid (HSD/D5%) were stated at 4ml/kg/hr following the initial 2 boluses but the child arrested (asystole), dying 4.5 hours after admission, despite unsuccessful attempts at resuscitation.  **Attending clinician’s diagnosis**: Patient died after 4.5 hours, was severely ill (severe malnutrition and malaria) with acidosis and hypoxia. Death likely due to severity of illness and multiple organ failure.  **End-point review:** The patient died from metabolic derangement and acute kidney failure in severe malnutrition not related to fluid overload. Classified as an early death. | **Group 1**  **(bolus + maintenance)** |
| **AFR 407**  Fatal | **Unrelated**  1/ Not related to bolus fluid and volume administered  Fatality related to severity of illness, acute renal and multiple organ failure | **Clinical History and admission findings**  A severely malnourished child weighing 5.3kg (WHZ-score > - 3) was admitted in shock (i.e. cold peripheries with a temperature gradient, weak and rapid radial pulse and CRT of 3 seconds) with a 1-week history of diarrhoea, fever and difficulty breathing.  Clinical examination at admission: The patient had prostration, relative bradycardia (heart rate 107 beats/min), relative hypopnea (respiratory rate 26 breaths/min), respiratory distress with deep breathing and chest wall in-drawing (oxygen saturation 88 % in room air), generalized oedema (kwashiorkor), mild liver enlargement (2cm below the right costal margin) but not in heart failure (clear chest on auscultation, no gallop cardiac rhythm, no distended neck veins).  Admission blood tests: Severe hyponatraemia (sodium 119mmol/L) and hypokalaemia (potassium 2.2mmol/L), elevated creatinine 110μmol/L, blood glucose 12.4 mmol/l, lactate 2.9 mmol/L. Marked leukocytosis (white blood cell count of 36,900/μL), thrombocytopenia (platelet count of 61,000/μL), haemoglobin 7.9 g/dl. Positive malaria rapid diagnostic test, and negative HIV test. BNP 83.2pg/ml and troponin I level was 0.  Initial treatment: Supplemental oxygen, fluid resuscitation with two boluses (RL, 15ml/kg/hr).  Intravenous antibiotics (ampicillin and gentamicin), anti-malarial treatment.  Echocardiographic findings: Fractional shortening (FS) before bolus fluid was 14% and rose to 22% after bolus fluid. Inferior vena caval collapsibility index (IVCCI) unexpectedly increased from 27% before bolus fluid to 37% after bolus fluid. The systemic vascular resistance index (SVRI) was high 1,673 dscm^-5^/m^2^ before bolus fluid and reduced slightly to 1,254 dscm^-5^/m^2^ after bolus fluid while the stroke volume index (SVI) increased from 18ml/m^2^ before bolus fluid to 29ml/m^2^ after bolus fluid.  Clinical progress:  After showing no initial improvement with the 2 boluses (RL, 15ml/kg/hr) the patient received blood transfusion of 10ml/kg (as per WHO recommendations) over 2 hours. Thereafter received maintenance fluid at 4ml/kg/hr. The clinical course was relatively short, the patient died after 8 hours; was oliguric, passed 1ml/kg/hr of loose watery stools (by diaper weights).  **Attending clinician’s diagnosis**: Patient died 8 hours after admission. Death likely due to severity of illness and multiple organ failure.  **End-point review:** The patient died from metabolic derangement and acute kidney failure in severe malnutrition not related to fluid overload. Classified as an early death. | **Group 1**  **(bolus + maintenance)** |
| **AFR 408**  Fatal | **Unrelated**  1/ Not related to bolus fluid and volume administered  Fatality related to severity of illness, acute renal failure and possible aspiration | **Clinical History and admission findings**  A severely malnourished child weighing 5 kg (WHZ-score > -4; MUAC 8.5cm) had a history of ill health for the past 1 month with unresolving diarrhoea and common cold.  Presented in respiratory distress and shock at admission (i.e. cold peripheries with a temperature gradient, weak and rapid radial pulse) CRT was however 2 seconds.  Clinical examination at admission: The patient had prostration, severe dehydration with a prolonged return of skin pinch (4-seconds), relative bradycardia (heart rate 106 beats/min), tachypnoea (respiratory rate 44 breaths/min), had bilateral coarse crackles on chest auscultation (severe pneumonia) but no signs of heart failure (i.e. no gallop rhythm or distension of neck veins).  Admission blood tests: Sodium 131mmol/L, hypokalaemia (potassium 2.5mmol/L), elevated creatinine 164μmol/L, blood glucose 6.1 mmol/L, lactate < 1.0 mmol/L, haemoglobin 7.4 g/dL and white blood cell count 10,600/μL. Positive malaria rapid diagnostic test but HIV testing was negative. BNP 1,637.8pg/ml and troponin I level was 0.  Initial treatment: Fluid resuscitation for shock with two boluses (RL, 15ml/kg/hr). Intravenous antibiotics (ceftriaxone was started due to prolonged history of illness) and anti-malarial treatment.  Echocardiographic findings: Fractional shortening (FS) before bolus fluid was 24% and rose to 27% after bolus fluid and remained unchanged (27%) at 24 hours. Inferior vena caval collapsibility index (IVCCI) reduced from 48% before bolus fluid to 38% immediately after bolus fluid. IVCCI increased marginally to 41% at 24 hours. The systemic vascular resistance index (SVRI) was normal for age 820 dscm^-5^/m^2^ before bolus fluid but rose slightly to 852 dscm^-5^/m^2^ after bolus fluid while the stroke volume index (SVI) increased as expected from 22ml/m^2^ before bolus fluid to 31ml/m^2^ after bolus fluid.  Clinical progress:  The patient was started on maintenance fluid (HSD/D5%) at 4ml/kg/hr after receiving 2 boluses (RL, 15ml/kg/hr). Haemoglobin level was found to have dropped to 5.4g/dL at 24 hours and the patient received transfusion of available blood at 7ml/kg over 2 hours which increased the haemoglobin level to 7.6g/dL. Over the short course of admission, the patient remained oliguric, passed 3ml/kg/hr of loose watery stools (by diaper weight), had persistent vomiting and was unable to tolerate any oral intake; died after 48 hours due to severity of illness.  **Attending clinician’s diagnosis**: Patient died 48 hours after admission. Death likely due to aspiration of vomitus.  **End-point review:** The patient died from severity of illness with acute renal failure in severe malnutrition with possible aspiration; unrelated to fluid overload. Classified as a late death. | **Group 1**  **(bolus + maintenance)** |
| **AFR 409**  Non-fatal (absconded treatment) | **Unrelated**  1/ Not related to bolus fluid and volume administered  Patient absconded after 4 days in hospital | **Clinical History and admission findings**  A severely malnourished child weighing 5.9 kg (WHZ-score > -4; MUAC 12cm) was admitted in shock (i.e. cold peripheries with a temperature gradient and prostration) with a history of diarrhoea, and vomiting.  Clinical examination at admission: The patient had relative bradycardia (heart rate 79 beats/min), CRT 2 seconds, respiratory distress with deep breathing and chest wall in-drawing (respiratory rate 26 breaths/min; oxygen saturation 96 %), oedematous with hair and skin changes, clear chest on auscultation and no signs of heart failure (i.e. no gallop rhythm or distension of neck veins).  Admission blood tests sodium 135mmol/L, hypokalaemia (potassium 2.3mmol/L), creatinine 67μmol/L, blood glucose 8.0 mmol/l, lactate 0.8 mmol/L, haemoglobin 9.1 g/dL and leukocytosis (white blood cell count 24,100/μL). Malaria rapid diagnostic test and HIV test were both negative. BNP 281.0pg/ml and troponin I level was 0.  Initial treatment Fluid resuscitation with 2 boluses (RL, 15ml/kg/hr). : Intravenous antibiotic (ceftriaxone due to markedly high white cell count).  Echocardiographic findings: Fractional shortening (FS) remained unchanged after bolus fluid (31%). Inferior vena caval collapsibility index (IVCCI) showed an unexpected gradual rise from 30% before bolus fluid to 39% immediately after bolus fluid and 49% at 24 hours. The systemic vascular resistance index (SVRI) was high 1,995 dscm^-5^/m^2^ before bolus fluid and decreased slightly to 1,539 dscm^-5^/m^2^ after bolus fluid while the stroke volume index (SVI) remained unchanged after bolus fluid 45ml/m^2^.  Clinical progress:  After the bolus, the patient received maintenance fluid (HSD/D5%) at 4ml/kg/hr for 48 hours as was unable to tolerate oral intake and had persistent vomiting but had good urine output of 7.8ml/kg/hr.  **Attending clinician’s diagnosis**: Patient absconded hospital 4 days after admission and died at home from severe malnutrition and likely complication of overwhelming sepsis.  **End-point review:** The patient was quite sick with severe malnutrition and septic skin lesions but absconded treatment from the hospital. SAE is unrelated to fluid overload or treatment received. | **Group 1**  **(bolus + maintenance)** |
| **AFR 412**  Non-fatal (absconded) | **Unrelated**  1/ Not related to bolus fluid and volume administered  Patient absconded treatment at the end of the third day | **Clinical History and admission findings**  A severely malnourished child weighing 6.0 kg (WHZ-score > -3; MUAC 12.3cm) presented with an acute history of diarrhoea and vomiting.  Admitted in shock (i.e. cold peripheries with a temperature gradient and prostrated).  Clinical examination at admission: The patient was severely wasted (marasmic), very sick and irritable, severe tachycardia (heart rate 181 beats/min), severe respiratory distress with Kussmaul’s breathing and chest wall in-drawing (oxygen saturation 82 %) and severe tachypnoea (respiratory rate 55 breaths/min). However, there were no signs of heart failure (i.e. clear chest on auscultation, no gallop rhythm and neck veins not distended).  Admission blood tests: hyponatraemia (sodium 129mmol/L), potassium 5.9mmol/L, elevated creatinine 171μmo/L, blood glucose 4.7 mmol/l and lactate 3.8 mmol/L. Marked leukocytosis (white blood cell count 67,000/μL) and haemoglobin 5.9 g/dL . Malaria rapid diagnostic test and HIV test were both negative. BNP 117.4pg/ml and troponin I level was 0.  Initial treatment: Slow rehydration over 5 hours (RL, 10ml/kg/hr) then started on oral rehydration solution for malnutrition (ReSoMal). The patient also received intravenous antibiotics (i.e. ampicillin and gentamicin).  Echocardiographic findings: Fractional shortening (FS) increased from 21% to 25% after fluid rehydration and remained unchanged by 24 hours. Inferior vena caval collapsibility index (IVCCI) gradually reduced from 59% to 38% after fluid rehydration as expected. The systemic vascular resistance index (SVRI) was markedly high at admission (i.e. 2,064 dscm^-5^/m^2^) and reduced slightly to 1,762 dscm^-5^/m^2^ after fluid rehydration. The stroke volume index (SVI) remained unchanged at 30ml/m^2^ before and after fluid rehydration.  Clinical progress:  Due to the low haemoglobin at admission, the patient also received blood transfusion of 8ml/kg/hr over 2 hours when blood was available 7 hours into admission but had persistent vomiting and diarrhoea (273mls by diaper weights). This patient was fairly sick, severely malnourished and further complicated by uncooperative guardians who had initially attempted to resist blood transfusion. The patient absconded treatment at the end of the third day into admission while still very sick.  **Attending clinician’s diagnosis**: Patient absconded hospital at the end of the 3^rd^ days after admission and reportedly died 5 days later in a private hospital facility. Mortality likely due to severity of illness.  **End-point review:** The patient was severely unwell and malnourished but absconded. SAE is unrelated to fluid overload or treatment received and patient could have likely succumbed to partially treated sepsis while seeking treatment at another hospital. | **Group 2**  **(rehydration-only)** |
| **AFR 413**  Fatal | **Unrelated**  1/ Not related to bolus fluid and volume administered  Fatality related to severity of illness and rising acidosis | **Clinical History and admission findings**  A severely malnourished child weighing 10 kg (WHZ-score > -3; MUAC 11.3cm) was admitted in shock (i.e. prostrated, cold peripheries with a temperature gradient and a weak pulse) with a history of diarrhoea and vomiting.  On physical examination, the patient was severely malnourished, oedematous (kwashiorkor), tachycardic **(**heart rate 160 beats/min), CRT 2 seconds, had severe respiratory distress (i.e. Kussmaul’s breathing and chest wall in-drawing), oxygen saturation 94 %, tachypnoea (respiratory rate 60 breaths/min), prolonged return of skin pinch > 3 seconds). There were right sided crackles anteriorly (pneumonia) but no gallop rhythm and no distended neck veins suggestive of heart failure.  Admission blood tests: severe hyponatraemia (sodium 123mmol/L), hypokalaemia (potassium 1.8mmol/L), elevated creatinine (114μmol/L), blood glucose 2.2 mmol/L, lactate 3.6 mmol/L. Leukocytosis (white blood cell count 20,100/μL) and haemoglobin 9.2 g/dL. Positive malaria rapid diagnostic test but HIV test was not done. BNP 151.2pg/ml and troponin I 369.6pg/ml.  Initial treatment: Slow rehydration over 5 hours (RL, 10ml/kg/hr) followed by maintenance fluid (HSD/D5%, 4ml/kg/hr) as unable to tolerate oral treatment. The patient received intravenous antibiotics (ampicillin and gentamicin) and anti-malarial treatment.  Echocardiographic findings: Fractional shortening (FS) increased from 34% to 38% after fluid rehydration. Inferior vena caval collapsibility index (IVCCI) unexpectedly increased from 28% to 34% after fluid rehydration. The systemic vascular resistance index (SVRI) was high 1,265 dscm^-5^/m^2^) and marginally reduced to 1,072 dscm^-5^/m^2^ after fluid rehydration. The stroke volume index (SVI) increased from 32ml/m^2^ to 40 ml/m^2^ after fluid rehydration.  Clinical progress:  The patient improved following rehydration with extremities warming up but was unable to take orally, still had tachycardia (heart rate 168 beats/min) and was started on maintenance fluid (HSD/D5%, 4ml/kg/hr). The child passed away 12 hours into admission following asystole and unsuccessful resuscitation with worsening acidosis (lactate 4.3mmol/L) and oliguria (urine output 0.5ml/kg/hr).  **Attending clinician’s diagnosis**: Patient died 12 hours after admission with worsening acidosis and severe illness as likely causes of death.  **End-point review:** The patient died of acute renal failure and severe malnutrition. Mortality not due to fluid overload. Classified as an early death. | **Group 2**  **(rehydration-only)** |
| **AFR 414**  Fatal | **Unrelated**  1/ Not related to bolus fluid and volume administered  Fatality related to severity of illness, respiratory arrest and acidosis | **Clinical History and admission findings**  A severely malnourished child weighing 8.5 kg (WHZ-score > -3; MUAC 12cm) was admitted in shock (i.e. prostration, cold peripheries with a temperature gradient and a prolonged CRT > 3 seconds) with a history of diarrhoea and difficulty breathing leading to admission.  Clinical examination at admission: Severely malnourished with generalized oedema (kwashiorkor), respiratory distress (i.e. deep breathing and chest wall in-drawing), tachypnoea ( respiratory rate 44 breaths/min), but good oxygen saturation (98%) with no signs of heart failure (i.e. clear chest on auscultation, no gallop rhythm, no distended neck veins) and heart rate 115 beats/min.  Admission blood tests: Hyponatraemia (sodium 122mmol/L), hypokalaemia (potassium 2.2mmol/L), creatinine 72μmol/L, hypoglycaemia (blood glucose 2.2 mmol/L), and lactate 2.1 mmol/L. Haemoglobin 5.3 g/dL, white blood cell count 9,700/μL, thrombocytopenia (platelets 52,000/μL. Positive malaria rapid diagnostic test HIV test was not done. BNP 110.1pg/ml and troponin I level was 0.  Initial treatment: slow rehydration over 3 hours (RL, 10ml/kg/hr) and switched to oral rehydration solution for malnutrition (ReSoMal) as tolerated. The patient also commenced intravenous antibiotics (ampicillin and gentamicin), anti-malarial treatment, a received glucose correction (5ml/kg of 10% dextrose) for hypoglycaemia.  Echocardiographic findings: Fractional shortening (FS) marginally decreased from 33% to 31% after fluid rehydration. Inferior vena caval collapsibility index (IVCCI) remained unchanged after fluid rehydration (21%). The systemic vascular resistance index (SVRI) marginally increased from 701 dscm^-5^/m^2^ to 749 dscm^-5^/m^2^ after fluid rehydration. The stroke volume index (SVI) increased from 45ml/m^2^ to 60 ml/m^2^ after fluid rehydration.  Clinical progress:  The patient showed initial improvement with slow rehydration in the first 3 hours, woke up and was able to take orally. The child was started on F75 therapeutic milk treatment for malnutrition and also received a blood transfusion of 9ml/kg/hr over 2 hours which improved haemoglobin level to 9.7g/dL. Urine output was good (3ml/kg/hr), however, the patient still continues passing loose watery stools (3ml/kg/hr over 24 hours, by diaper weights). Despite initial improvement and correction of electrolyte abnormalities (sodium 135mmol/L and potassium 4mmol/L), the patient developed severe deep breathing, decreasing oxygen saturations (67%) and lactic acidosis (4mmol/L) on day 4 of admission.  **Attending clinician’s diagnosis**: Patient died 4 days after admission due to respiratory arrest non-responsive to resuscitation.  **End-point review:** The patient died from respiratory complication but did not have any signs of volume overload (no crackles suggestive of pulmonary oedema or hepatomegaly/neck vein distension), therefore mortality is unrelated to fluids. Classified as a late death. | **Group 2**  **(rehydration-only)** |
| **AFR 415**  Fatal | **Unrelated**  1/ Not related to bolus fluid and volume administered  Fatality related to severity of illness, respiratory arrest | **Clinical History and admission findings**  A severely malnourished child weighing 7 kg (WHZ-score > -3; MUAC 10.2cm), with bipedal oedema (kwashiorkor) was admitted in coma and in respiratory arrest with shock (i.e. cold peripheries with a temperature gradient, a very weak pulse) and a 3-week history of diarrhoea, vomiting.  Clinical examination at admission: The patient was comatose, in respiratory arrest, bradycardic (heart rate 72 beats/min), CRT > 2 seconds, severely dehydrated (i.e. prolonged return of skin pinch, 6 seconds).  Admission blood tests: Severe hyponatraemia (sodium 122mmol/L), hypokalaemia (potassium 2 mmol/L), elevated creatinine (108μmol/L), severe hypoglycaemia (blood glucose 1.4 mmol/L) and lactate 1.6 mmol/L, haemoglobin 9.7 g/dL, white blood cell count 10,300/μL. Malaria rapid diagnostic test and HIV test were both negative. BNP 73.1pg/ml and troponin I level was 0.  Initial treatment: Resuscitation from respiratory arrest, blood glucose correction (5ml/kg of 10% dextrose), intravenous antibiotics (ceftriaxone), fluid rehydration (RL, 10ml/kg/hr) over 3 hours.  Echocardiographic findings: Fractional shortening (FS) remained unchanged after fluid rehydration (31%). Inferior vena caval collapsibility index (IVCCI) reduced as expected from 39% to 28% after fluid rehydration. The systemic vascular resistance index (SVRI) was high before (1,093 dscm^-5^/m^2^) and after (1,070 dscm^-5^/m^2^) fluid rehydration while the stroke volume index (SVI) remained unchanged (31ml/m^2^) after fluid rehydration.  Clinical progress:  The patient responded to initial resuscitation and received IV maintenance fluid (HSD/D5%, 4ml/kg/hr) following the fluid rehydration, urine output was 3.2 ml/kg/hr, but the electrolyte abnormalities persisted despite treatment (sodium 124mmol/L, potassium 1.6mmol/L) and creatinine remained high 149μmol/L. 20 hours into admission, the patient went into respiratory arrest and resuscitation was not successful.  **Attending clinician’s diagnosis**: Patient died 20 hours after admission due to respiratory arrest non-responsive to resuscitation.  **End-point review:** The patient died from persisting metabolic and electrolyte abnormalities in severe malnutrition and terminal respiratory arrest. Mortality is unrelated to fluids. Classified as an early death. | **Group 2**  **(rehydration-only)** |
| **AFR 416**  Fatal | **Unrelated**  1/ Not related to bolus fluid and volume administered  Fatality related to severe illness with multiple organ failure leading to cardio-respiratory arrest | **Clinical History and admission findings**  A severely malnourished child weighing 6 kg (WHZ-score > -3; MUAC 7cm) and oedematous (kwashiorkor) had a history of diarrhoea and cough for 14 days prior to admission.  At admission, the patient was comatose and in shock (i.e. cold peripheries with a temperature gradient and a very weak pulse, prolonged CRT > 5 seconds). Had relative hypopnea (respiratory rate 20 breaths/min) with respiratory distress (deep breathing, chest wall in-drawing and un-recordable oxygen saturation), heart rate 144 beats/min and prolonged return of skin pinch (4 seconds).  Admission blood tests: Hyponatraemia (sodium 125 mmol/L, hyperkalaemia (potassium 6.5 mmol/L), hypoglycaemic (blood glucose 1.3 mmol/L), lactate 2.2 mmol/L, haemoglobin 9.8 g/dl, white blood cell count 8,800/μL, creatinine 59μmol/L. Malaria rapid diagnostic test and HIV test were both negative. BNP 168.0pg/ml and troponin I 915.7pg/ml.  Initial treatment: Blood glucose correction (5ml/kg of 10% dextrose), intravenous antibiotics (ceftriaxone) and slow fluid rehydration (RL, 10ml/kg/hr).  Echocardiographic findings: At admission, the fractional shortening (FS) was 32%, the systemic vascular resistance index (SVRI) was high 1,207 dscm^-5^/m^2^ and the stroke volume index (SVI) was 35ml/m^2^. Assessment of the inferior vena cava for quantification of the collapsibility index (IVCCI) was not possible as the IVC was barely visible on echo assessment. The patient was critically unwell, had a very brief clinical course and died after 4 hours.  Clinical progress:  The patient showed no improvement and went into cardio-respiratory 4 hours into admission. Resuscitation attempt was unsuccessful.  **Attending clinician’s diagnosis**: Patient died 4 hours after admission due to severity of illness leading to multiple organ failure and cardio-respiratory arrest.  **End-point review:** The patient died from cardio-respiratory arrest resulting from severe illness and multiple organ failure. There was no evidence of volume overload and mortality is unrelated to fluids administered. Classified as an early death. | **Group 2**  **(rehydration-only)** |
| **AFR 417**  Fatal | **Unrelated**  1/ Not related to bolus fluid and volume administered  Fatality related to severe illness (i.e. shock in severe malnutrition with HIV and complications of severe pneumonia and diarrhoea) | **Clinical History and admission findings**  A severely malnourished child weighing 4 kg (WHZ-score -3; MUAC 8.3cm) was admitted in shock (i.e. had prostration, cold peripheries with a temperature gradient, weak pulse and CRT > 4 seconds) following a 7-day history of diarrhoea and fever.  Physical examination at admission: The patient had severe respiratory distress (Kussmaul’s breathing, respiratory rate 44 breaths/min, profound hypoxia oxygen saturation of 32% and central cyanosis), heart rate 144 beats/min and prolonged return of skin pinch (5 seconds). Coarse crackles in the chest on auscultation but no signs of heart failure/overload (no gallop rhythm or distension of neck veins).  Admission blood tests: Very severe hyponatraemia (sodium 119 mmol/L), hypokalaemia (potassium 2.6 mmol/L), creatinine 24μmol/L , hypoglycaemia (blood glucose 1.9 mmol/L), and lactate 1.2 mmol/L, Leukocytopenia (white blood cell count 2,800/μL), thrombocytopenia (platelets 54,000/μL) and haemoglobin 9.2 g/dL, Negative malaria rapid diagnostic test; HIV test was positive. BNP 213.9pg/ml and troponin I level was 0.  Initial treatment: Blood glucose correction (5ml/kg of 10% dextrose), intravenous antibiotics (ceftriaxone) and slow fluid rehydration (RL, 10ml/kg/hr).  Echocardiographic findings: At admission, the fractional shortening (FS) was 26%, the inferior vena cava collapsibility index (IVCCI) was 49%, the systemic vascular resistance index (SVRI) was high 1,155 dscm^-5^/m^2^ and the stroke volume index (SVI) was low 18ml/m^2^. The patient had a very short clinical course and died 2 hours after admission.  Clinical progress:  The patient’s condition rapidly deteriorated with worsening acidosis (lactate 5.4mmol/L) and died after 2 hours while undergoing resuscitation.  **Attending clinician’s diagnosis**: Early/immediate death due to shock in severe malnutrition and HIV illness with very severe pneumonia and diarrhoea.  **End-point review:** The patient died from shock and complications of untreated HIV illness (severe pneumonia and diarrhoea). Mortality not related to fluids given and classified as an early death. | **Group 2**  **(rehydration-only)** |
| **AFR 419**  Non-fatal  (absconded) | **Unrelated**  1/ Not related to bolus fluid and volume administered  Patient absconded after 6 days in hospital | **Clinical History and admission findings**  A child weighing 5.0 kg (WHZ-score -3; MUAC 11cm); with severe wasting (marasmus) presented with a short history of diarrhoea and was admitted to hospital in hypovolaemic shock (i.e. had prostration, cold peripheries with a temperature gradient and a weak pulse).  Physical examination: The patient was severely wasted, had a prolonged return of skin pinch (4 seconds), respiratory distress with chest in-drawing, respiratory rate 30 breaths/min, clear chest on auscultation with oxygen saturation at 98%, heart rate 109 beats/min and not in heart failure (i.e. no distension of neck veins and no gallop heart rhythm).  Admission blood tests: Haemoglobin 10.9g/dL, lactate 1.9mmol/L, malaria rapid diagnostic test and HIV test were both negative. BNP 191pg/ml and troponin I level was 0.  Initial treatment: The patient received slow rehydration over 3 hours (RL, 10ml/kg/hr) then started on oral rehydration solution for malnutrition (ReSoMal) via naso-gastric tube. Also commenced intravenous antibiotics (ampicillin and gentamicin).  Echocardiographic findings: Fractional shortening (FS) marginally increased from 34% to 36% after fluid rehydration but reduced to 29% by 24 hours. Inferior vena caval collapsibility index (IVCCI) unexpectedly increased from 28% to 36% after fluid rehydration but dropped to 23% by 24 hours. The systemic vascular resistance index (SVRI) was high 1,895 dscm^-5^/m^2^ and reduced slightly to 1,548 dscm^-5^/m^2^ after fluid rehydration then rose to 2,284 dscm^-5^/m^2^ by 24 hours. The stroke volume index (SVI) increased gradually as expected from 32ml/m^2^ to 41 ml/m^2^ after fluid rehydration.  Clinical progress:  The patient showed remarkable improvement clinically while on treatment, had a good urine output 2.9ml/kg/hr but very high volume of diarrhoea persisted (average volume of 669ml/day, by diaper weights). The patient absconded treatment 6 days after admission while on F75 therapeutic milk formulation for severe malnutrition.  **Attending clinician’s diagnosis**: Patient absconded hospital 6 days after admission while on treatment for severe malnutrition and was lost to follow-up.  **End-point review:** The patient was severely ill at admission but responded well to treatment. Absconded while still on treatment for severe malnutrition. SAE is unrelated to fluid rehydration given. | **Group 2**  **(rehydration-only)** |
